# Biocultural diversity of common walnut (*Juglans regia* L.) and sweet chestnut (*Castanea sativa* Mill.) across Eurasia

**DOI:** 10.1002/ece3.6761

**Published:** 2020-09-24

**Authors:** Paola Pollegioni, Stefano Del Lungo, Ruth Müller, Keith E. Woeste, Francesca Chiocchini, Jo Clark, Gabriel E. Hemery, Sergio Mapelli, Fiorella Villani, Maria Emilia Malvolti, Claudia Mattioni

**Affiliations:** ^1^ Research Institute on Terrestrial Ecosystems National Research Council Porano Terni Italy; ^2^ The Institute of Cultural Heritage Science National Research Council Tito Scalo Potenza Italy; ^3^ Unit Entomology Department of Biomedical Sciences Institute of Tropical Medicine Antwerp Belgium; ^4^ Hardwood Tree Improvement and Regeneration Center Department of Forestry and Natural Resources U.S.D.A. Forest Service Purdue University West Lafayette IN USA; ^5^ Future Tree Trust Stroud Gloucestershire UK; ^6^ Sylva Foundation Oxfordshire UK; ^7^ Institute of Agricultural Biology and Biotechnology National Research Council Milan Italy

**Keywords:** anthropogenic processes, common walnut, human linguistic diversity, population genetics, sweet chestnut

## Abstract

A biocultural diversity approach integrates plant biology and germplasm dispersal processes with human cultural diversity. An increasing number of studies have identified cultural factors and ethnolinguistic barriers as the main drivers of the genetic diversity in crop plants. Little is known about how anthropogenic processes have affected the evolution of tree crops over the entire time scale of their interaction with humans. In Asia and the Mediterranean, common walnut (*Juglans regia* L.) and sweet chestnut (*Castanea sativa* Mill.) have been economically and culturally important crops for millennia; there, in ancient times, they were invested with symbolic and religious significance. In this study, we detected a partial geographic congruence between the ethno‐linguistic repartition of human communities, the distribution of major cognitive sets of word‐related terms, and the inferred genetic clusters of common walnut and sweet chestnut populations across Eurasia. Our data indicated that isolation by distance processes, landscape heterogeneity and cultural boundaries might have promoted simultaneously human language diversification and walnut/chestnut differentiation across the same geographic macro‐regions. Hotspots of common walnut and sweet chestnut genetic diversity were associated with areas of linguistic enrichment in the Himalayas, Trans‐Caucasus, and Pyrenees Mountains, where common walnuts and sweet chestnuts had sustained ties to human culture since the Early Bronze Age. Our multidisciplinary approach supported the indirect and direct role of humans in shaping walnut and chestnut diversity across Eurasia from the EBA (e.g., Persian Empire and Greek–Roman colonization) until the first evidence of active selection and clonal propagation by grafting of both species. Our findings highlighted the benefit of an efficient integration of the relevant cultural factors in the classical genome (G) × environmental (E) model and the urgency of a systematic application of the biocultural diversity concept in the reconstruction of the evolutionary history of tree species.

## Graphical abstract

Little is known about how anthropogenic processes have affected the evolution of tree species with a long‐time‐scale history of human utilization such as common walnut (*Juglans regia* L.) and sweet chestnut (*Castanea sativa* Mill.). In this study, we evaluated the impact of isolation by distance processes, landscape heterogeneity, and cultural boundaries among human communities on shaping genetic resources of walnut and chestnut across Eurasia. Our findings highlighted the benefit of an efficient integration of the relevant cultural factors in the classical genome (G) × environmental (E) model and the urgency of a systematic application of the biocultural diversity concept in the reconstruction of evolutionary history of tree species

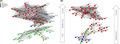

## 1 INTRODUCTION

The field of plant biocultural diversity is a dynamic and integrative approach combining data from plant biology and germplasm dispersal processes with information about human cultural and linguistic diversity. These factors are interrelated because plants, especially crops, coevolved with humans in a complex socio‐ecological system at scales from the global to the local (Gavin et al., 2015; Maffi, 2005). This holistic view emerged over the last decade, connecting the relationships between humans and the natural world and other social factors with the development of new conservation strategies in landscape genetics and urban ecology (Elands et al., 2019). Leclerc and Coppens d’Eeckenbrugge (2012) expanded the classical genome (G) × environmental (E) formula (G × E) to a three‐way model (G × E × S) in which plant species were considered social objects (S). Indeed, marriage exchanges, traditional ethnic customs, and social networks have been identified as the main drivers preserving and shaping the genetic resources in crop species invested with esthetic, ethnobotanic and religious significance. Examples include cereals in the Yunnan Province of China (Xu et al., 2014), cassava in Gabon (Delêtre, McKey, & Hodkinson, 2011), sorghum in Kenya (Labeyrie et al., 2014), and maize in Mexico (Orozco‐Ramírez, Ross‐Ibarra, Santacruz‐Varela, & Brush, 2016). These researchers investigated farming communities at local‐national level, but did not provide a comparative and broader overview of the plant biocultural diversity.

A few pioneering studies shed light on the cultural forces affecting plant diversity and seed dispersal routes at a continental scale. A strong geographic coincidence between ethnolinguistic boundaries, used as a proxy for human cultural differences, and the population genetic structure of plants has been detected for sorghum and pearl millet in Africa (Naino Jika et al., 2017; Westengen et al., 2014), banana and sweet potato in Oceania (Perrier et al., 2011; Roullier, Benoit, McKey, & Lebot, 2013), and baobab in Australia (Rangan et al., 2015). In these studies, the integration of ethnobotanical evidence with genetic and linguistic data (e.g., movement of word‐related terms) clarified the complex history of human manipulation and dispersal of crop plants and the role of cultural anthropology on crop genetic diversification. Little is known about how ethnolinguistic diversity and cultural–historical processes affected the evolution and distribution of tree species with a long history of human utilization in agroforestry, such as common walnut (*Juglans regia* L.) and sweet chestnut (*Castanea sativa* Mill.).

Both sweet chestnut and common walnut are economically important, monoecious, dichogamous, long‐lived, perennial trees cultivated worldwide for high‐quality wood, edible nuts, and several secondary products. Pollination is described as anemophilous for both species but also entomophilous for chestnut. For millennia, both species have been endowed with symbolic and religious significance by societies in Asia and the Mediterranean which fully incorporated them into their cultures (Conedera, Krebs, Tinner, Pradella, & Torriani, 2004; Vahdati, 2014). In the last two decades, the history of common walnut and sweet chestnut has emerged as a complex interaction of biogeographic and human forces (Figure S1). Genetic and fossil evidence demonstrated that the actual distribution of common walnut in Eurasia resulted from the combined effects of expansion/contraction from multiple refugia, ranging from Central Asia, through the Caucasus to the Balkans and Western Europe, after the Last Glacial Maximum (Pollegioni et al., 2017). Similarly, Krebs, Pezzatti, Beffa, Tinner, and Conedera (2019) proposed a precultivation spontaneous spread of sweet chestnut from macro‐refugia located in Trans‐caucasia, where the tree species played a prominent role in the forest vegetation, and in the Italian and Iberian Peninsulas where the species probably persisted in scattered pockets of favorable habitats. The human spread of both species became dominant in the late Holocene, and the possibility of early cultivation attempts and seed exchanges during the Early Bronze Age (EBA) in the Balkan–Anatolian–Caucasian circuit is a matter of long‐standing debate (Bottema, 2000; Krebs et al., 2019). Despite this information, the onset of common walnut and sweet chestnut arboriculture is generally attested from 2,750–1,900 BP in Europe, coincident with the beginning of Greek and Roman dominance (Conedera et al., 2004). The Romans introduced both species across North‐Central Europe (Fig. S1), although no clear evidence of systematic planting exists. Nevertheless, Pollegioni et al. (2015) revealed that humans harvested and traded walnut along "corridors” such as the Silk Roads and the Persian Royal Road during the same time‐window, and what appeared to be native walnut stands were actually the result, at least in part, of ancient human efforts to modify the Asian landscape. The first evidence of active selection and clonal propagation by grafting was attested in Europe only from 15th to 18th centuries AD for sweet chestnut (Pereira‐Lorenzo et al., 2019) and in the last century for common walnut (Dehghan, Vahdati, Rezaee, & Hassani, 2009). We may therefore assume that long‐standing human contact and exclusive seed‐mediated propagation through the centuries have affected the genetic structure of common walnut and sweet chestnut natural populations across Eurasia until at least the Medieval period.

In this study, the analysis of two large and unique collections of *J. regia* (Pollegioni et al., 2017) and *C. sativa* (Mattioni et al., 2017) populations gave us the opportunity to provide a comprehensive and comparative view of the biocultural diversity of common walnut and sweet chestnut across Eurasia. Our objective was the integration of archeological, linguistic, and genetic data to address the role of landscape and ethno‐linguistic boundaries, used as a proxy of cultural similarities between human communities, on limiting and/or facilitating the gene flow of walnut and chestnut germplasm across Eurasia. We also sought to provide insight into the indirect and/or direct human‐mediated expansion of both species during historical eras such as the Aegean‐Anatolian EBA, the Persian Empire (starting from the Achaemenid phase, 2,450–2,280 YBP), and Greek–Roman colonization (≤2,550 BP). In particular, we aimed to infer (a) geographic coincidences between genetic boundaries among tree populations and languages repartition of human communities, (b) spatial congruence between the genetic richness of common walnut and sweet chestnut populations and linguistic human diversity in terms of associated word‐terms within language families of the native range, and (c) geographic overlaps between cognate sets of associated word‐terms and tree population genetic clusters.

## 2 MATERIALS AND METHODS

### 2.1 Genetic database for common walnut and sweet chestnut populations

This study makes use of two recently published datasets for displaying the genetic diversity of *J. regia* and *C. sativa* in their respective native range. The first study included 40 Asian autochthonous common walnut populations sampled from China, Kyrgyzstan, Uzbekistan, Tajikistan, Pakistan, Iran, Turkey, and Georgia and 51 European walnut populations sampled from Greece, Romania, Moldova, Hungary, Slovakia, Spain, France, and Italy, growing in thirteen mountain systems for a total of 91 populations and 2,008 genotypes. The second dataset comprised 73 sweet chestnut populations for a total of 1,608 wild chestnut trees sampled in 11 European (Spain, Portugal, France, England, Italy, Slovakia, Hungary, Romania, Bulgaria, Greece, and Russia) and 3 Asian (Turkey, Georgia, and Azerbaijan) countries (Table S1). All the sampling sites refer to natural or naturalized areas, excluding orchard or recent forest plantations. Both collections were genotyped using unlinked nuclear, neutral microsatellite (SSR) markers (fourteen and six loci in common walnut and sweet chestnut, respectively; Mattioni et al., 2017; Pollegioni et al., 2017).

### 2.2 Languages and human populations

Based on the Ethnologue website (Gordon, 2005), twenty‐two languages (Chinese‐Mandarin, Uyghur, Tibetan, Kyrgyz, Northern Uzbek, Tajiki, Urdu, Persian‐Iranian, Georgian, Azeri Turkish, Turkish, Russian, Greek, Bulgarian, Romanian, Hungarian, Slovak, Italian, French, Spanish, English, and Portuguese) were recorded in our sampled sites and classified into five linguistic phyla (Altaic, Indo‐European, Sino‐Tibetan, Kartvelian, and Uralic), twelve linguistic families (Turkic, Iranian, Sinitic, Tibeto‐Burman, Indo‐Aryan, Anatolian, Karto‐Zan, Hellenic, Balto‐Slavic, Germanic, Italic, and Finno‐Ugric), and thirteen linguistic subgroups (Western‐Turkic, Eastern‐Turkic, Southern‐Turkic, Oghuz‐Turkish, Western Iranian, Central Indo‐Aryan, East‐Slavic, South‐Slavic, Western‐Slavic, Attic‐Hellenic, Eastern‐Romance, Western Romance, and Anglo‐Frisian; Table S1).

Eight geographic areas were classified as sites with bilingual speakers. Uyghur is classified as an Eastern‐Turkic language currently spoken by 11 million people mainly living in the Xinjiang Province of North‐Western China. The urban areas of Xinjiang have recently faced major changes in their demographic and linguistic landscapes. Since the bilingual education policy was introduced in 2002, Chinese‐Mandarin language has been rapidly institutionalized (Smith Finley & Zang, 2015). The former multilingual pluralism of this region has been progressively replaced in favor of monolingual model. Similarly, standard Tibetan, along with Mandarin Chinese, is the official language spoken in the Tibet Region of South‐Western China (Gordon, 2005). Bakhmal is located in the Jizakh province of Central Uzbekistan bordering Tajikistan to the southeast and Chvigepse is placed in the North Caucasus region of Southern Russia. In these regions, Northern Uzbek and Russian are the official national languages, whereas Tajiki‐Persian and Georgian are currently spoken as a result of geographical proximity with Tajikistan and Georgia, respectively. Finally, Turkic speakers of Anatolia are descendants of indigenous Indo‐European farmers who adopted Turkic only from ~1,000 years BP. According to Hodoğlugil and Mahley (2012), Turkic‐speaking nomads, mainly Oghuz groups, spread away from their homeland in Central Asia and occupied the grassland in the interior of Asia Minor. These Turkic nomads, and later Ottomans, imposed their language on indigenous peoples and replaced Anatolian, an extinct branch of the Indo‐European family, by the *elite dominance* scenario.

### 2.3 Reconstructing walnut and chestnut proto‐words, inherited terms, loanwords, and open compound words

Linguistic terms for common walnut (Table S2) and sweet chestnut (Table S3) were collected from published sources and from the Language of the World Etymological Database (LWED) (http://starling.rinet.ru). If the etymological reconstruction was available, the proto‐word for walnut and chestnut forms, conventionally denoted with an asterisk (*) at the start of the word, was reported in the Dené‐Sino‐Caucasian (Basque, Proto_Burushaski, Proto‐North Caucasian, and Sino‐Tibetan), and Afro‐Asiatic and Eurasiatic (Dravidian, Kartvelian, Altaic, Indo‐European, and Uralic) Super‐Phylum. Despite the lack of written records, the etymological analysis of later attested words provided the opportunity to trace back through successive intermediate steps to the common ancestral word form in the ancestral reconstructed proto‐language. We considered linguistic terms as “inherited” when they were inherited from a proto‐language through nodes of phylogenic tree of more recent descendant languages following a vertical transmission. We classified linguistic terms as “loanwords” when words were borrowed from different language families and adopted in other languages by horizontal transmission. The identification of loanwords was further corroborated by archeological and historical data. We referred to a “cognate set” when the words have a common etymological origin sharing the same proto‐word. However, the cognates progressively changed their form and sometimes meaning over the course of the time (called false friends), but in most cases they have similar sounds (Campbell, 2013). In this study, we included already established cognate sets for walnut and chestnut word forms as proposed by The Languages of the World Etymological Database, part of the Tower of Babel project (LWED). Finally, we included open compound forms denoting walnut or chestnut made up of two words written separately but providing a unique meaning.

### 2.4 Genetic and linguistic data analysis

#### 2.4.1 Genetic structure of tree populations and language diversity in human communities

To explore the genetic relationships among 91 common walnut populations and 73 sweet chestnut populations in the native range (d_GEN_), we computed pairwise genetic differentiation with Jost's coefficient (Jost, 2008) using GenAlEx version 6.5 (Peakall & Smouse, 2012). Linguistic distances among human communities living in the sampling areas were calculated as simple dissimilarity indexes ranging from 0 to 4 according to the d_LAN_ matrix method described by Excoffier, Harding, Sokal, Pellegrini, and Sanchez‐Mazas (1991) and Belle and Barbujani (2007). The nonparametric Kruskal–Wallis test was used to detect statistical differences in the common walnut or sweet chestnut genetic differentiation among five human linguistic distance‐classes. Pairwise comparisons among linguistic classes for d_GEN_ values were performed based on post hoc Dunn's test using R‐ *dunn.test* package (https://CRAN.R‐project.org/package=dunn.test).

To estimate the genetic differentiation at both levels, among populations and among ethnolinguistic regions, we grouped common walnut and sweet chestnut genotypes according to their occurrence in the language phylum areas. Hierarchical analysis of molecular variance (AMOVA) was performed as implemented in Arlequin version 3.11 software (Excoffier, Laval, & Schneider, 2005), and statistical significance of Wright's *F‐*statistic estimators was tested using a nonparametric approach with 1,000 permutations. Furthermore, the spatial congruence between the genetic relationships among *J. regia* or *C. sativa* populations and the linguistic‐phylum patterns of human communities in the sampled sites was shown using a multivariate graph approach (EDENETWORKS v2.16, Kivela, Arnaud‐Haond, & Samarki, 2015). We constructed a minimum‐spanning tree plotting all populations (nodes) in a network graph with connections (edges) between all nodes. In the resulting graph, each edge was weighted according to its pairwise genetic distance and n populations were represented by n nodes with color equivalent to the language phylum spoken by human communities. Nodes were connected by the minimum number of edges necessary to minimize the overall genetic differentiation.

Partial Mantel tests of the genetic differentiation among common walnut and sweet chestnut populations (d_GEN_) versus human linguistic diversity (d_LAN_) with geographic distance as a covariate (d_GEO_) was used to test whether any statistical significance inferred by the AMOVA was a result of isolation by distance (IBD) (Smouse, Long, & Sokal, 1986). The p‐value for the z‐score of the Mantel association parameter was inferred using 5,000 permutations as implemented in ZT software (Bonnet & Van der Peer, 2002). Assuming a nonlinear distribution of sampling sites, we first tested for IBD by regressing [d_GEN_/(1 − d_GEN_)] pairwise values against the corresponding natural logarithm of geographic distances. Subsequently, we performed a partial Mantel test to calculate the partial correlation between linearized d_GEN_ values and human linguistic diversity after controlling for straight‐line geographic distance. Considering that partial Mantel tests showed inflated *type‐1* error rate in the presence of spatial autocorrelation (Guillot & Rousset, 2013), the influence of geographic distances and human linguistic diversity on d_GEN_ was also evaluated with a multiple regression on distance matrices approach using *MRM* function implemented in the R‐*ecodist* package (Goslee & Urban, 2007). The significance of regression coefficients and model *r^2^* was estimated using 5,000 permutations.

#### 2.4.2 Genetic richness of tree populations and word form‐related diversity in human communities

The level of genetic diversity was estimated for each common walnut and sweet chestnut population by computing the allelic richness (*Rs*) parameter using the rarefaction method as implemented in the HP‐Rare (Kalinowski, 2004). Following the procedure of Pollegioni et al. (2017) and Mattioni et al. (2017), the Inverse Distance Weighted (IDW) interpolation function implemented in the ArcGIS 9.3 software (ESRI, Redlands, Calif. USA) was used to display the geographic patterns of allelic richness (*Rs*) for all 91 common walnut and 73 sweet chestnut populations across Eurasia. For each sampling site, we calculated the number of common walnut and sweet chestnut linguistic forms recorded from published sources and from the LWED in terms of inherited terms, loanwords and open compound words. The nonparametric Kruskal–Wallis test was applied to compute statistical differences in the within‐population genetic variation *Rs* among classes of word‐term richness. Pairwise comparisons among word‐term classes for *Rs* values were performed based on post hoc Dunn's test.

#### 2.4.3 Genetic structure of tree populations and cognitive sets referred to walnut and chestnut terms

The geographic coincidences between genetic structure of walnut and chestnut populations and the distribution of the major cognitive sets referred to their respective word forms were examined. As reported in Pollegioni et al. (2017) and Mattioni et al. (2017), a fully Bayesian clustering approach as implemented in STRUCTURE 2.3.3 (Pritchard, Stephens, & Donnelly, 2000) was conducted to detect the most likely number of tree populations. After determining the most probable number of clusters (*K*), we derived two synthetic maps representing the genetic structure of common walnut and sweet chestnut in Eurasia. In addition, the absolute number of migrants exchanged between the inferred genetic clusters per generation (2Nm) was calculated using Arlequin version 3.11 software. The spatial distribution of walnut/ chestnut word‐terms and their proto‐words was compared to the inferred genetic clusters of *J. regia* and *C. sativa* populations across Eurasia. *Chi‐squared* tests were conducted to compute statistical differences in the distributions of the major cognate sets that referred to walnut and chestnut linguistic terms among the inferred genetic clusters.

## 3 RESULTS

### 3.1 Genetic differentiation of common walnut and sweet chestnut populations across language family areas

We observed a statistically significant positive trend between genetic distances (dGEN) among J. regia and C. sativa populations and linguistic distance (dLAN) among human communities living in the Eurasian sampling sites (Kruskal–Wallis tests: *p* < .0001). (Kruskal–Wallis tests: *p* < .0001). An increase of mean pairwise d_GEN_ was associated with an increase of mean d_LAN_ among human communities in both species, varying from d_GEN_ = 0.086 ± 0.003 (walnut) and d_GEN_ = 0.374 ± 0.01 (chestnut) for the category d_LAN_ = 0 (same language) to d_GEN_ = 0.393 ± 0.003 (walnut) and d_GEN_ = 0.693 ± 0.001 (chestnut) for the category d_LAN_ = 4 (different phyla) (Fig. S2). Furthermore, the genetic diversity of both species showed a significant isolation by distance pattern (IBD) in Eurasia (Table 1). The pairwise linearized genetic differentiation values and the natural logarithm of geographic distances (straight‐line distances in km) among sampling sites were in fact significantly correlated in common walnut (*r* = 0.737, *p* = .0002) and sweet chestnut (*r* = 0.569, *p* = .0002). Simple Mantel tests revealed that human linguistic diversity was also positively correlated with straight‐line geographic distances among common walnut (*r* = 0.739, *p* = .0002) and sweet chestnut (*r* = 0.567, *p* = .0002) sampling sites. Thus, the observed relationship between d_GEN_ and d_LAN_ matrices might have occurred in *J. regia* (*r* = 0.636, *p* = .0002) and *C. sativa* (*r* = 0.546, *p* = .0002) as a result of a common spatial component (Table 1). However, the partial correlation between human linguistic distances and genetic diversity remained significant but low even after the effect of d_GEO_ matrix was held constant in both species (walnut: *r* = 0.200, *p* = .0002; chestnut: *r* = 0.377, *p* = .0002). The MRM analysis indicated that the effects of geographic distance and human linguistic diversity on genetic tree divergence were significantly positive among common walnut populations (standardized partial regression coefficient: ß_GEO_ = 0.070, ß_LAN_ = 0.024, *p* < .0001) and sweet chestnut populations (ß_GEO_ = 0.087, ß_LAN_ = 0.057, *p* < .0001) (Table 1). The MRM model showed that geographic and language distance together explained 51.60% (*p* = .0002) and 39.7% (*p* = .0002) of common walnut and sweet chestnut genetic differentiation, respectively (Table 1).

**Table 1:** Correlation between genetic distances among common walnut/chestnut populations (d_GEN_) and human linguistic distances (d_LAN_)

	Common walnut		Sweet chestnut	
(A) Mantel test^a^	Correlation coefficient *(r)* ^b^	Proportion of variance explained *(r^2^)*	Correlation coefficient *(r)* ^b^	Proportion of variance explained *(r^2^)*
d_GEN_ × d_GEO_	0.737***	0.543	0.569***	0.324
d_GEN_ × d_LAN_	0.636***	0.404	0.546***	0.298
d_GEO_ × d_LAN_	0.739***	0.546	0.567***	0.321
(d_GEN_ × d_LAN_) •d_GEO_	0.200***	0.040	0.377***	0.142

(B) MRM^a^	Coefficient of Regression (β)^c^	*r* ^2^	Coefficient of Regression (β)^c^	*r* ^2^
Intercept	−0.291***	0.561***	−0.178	0.397***
D_GEO_	0.070***		0.087***	
D_LAN_	0.024***		0.057***	

^a^ (A) Simple and partial Mantel tests (Smouse et al., 1987) and (B) multiple regression model analysis of genetic (d_GEN_) on geographic (d_GEO_) and linguistic (d_LAN_) matrices.

^b^ Significance of *r* values was tested using 5,000 permutations as implemented in ZT software (Bonnet & Van der Peer, 2002): *p < .05; **p < .01, and ***p < .001.

^c^ P values are based on 5,000 permutations as implemented in R Ecodist package (Goslee & Urban, 2007): *p < .05; **p < .01; and ***p < .001.

**Figure 1:**
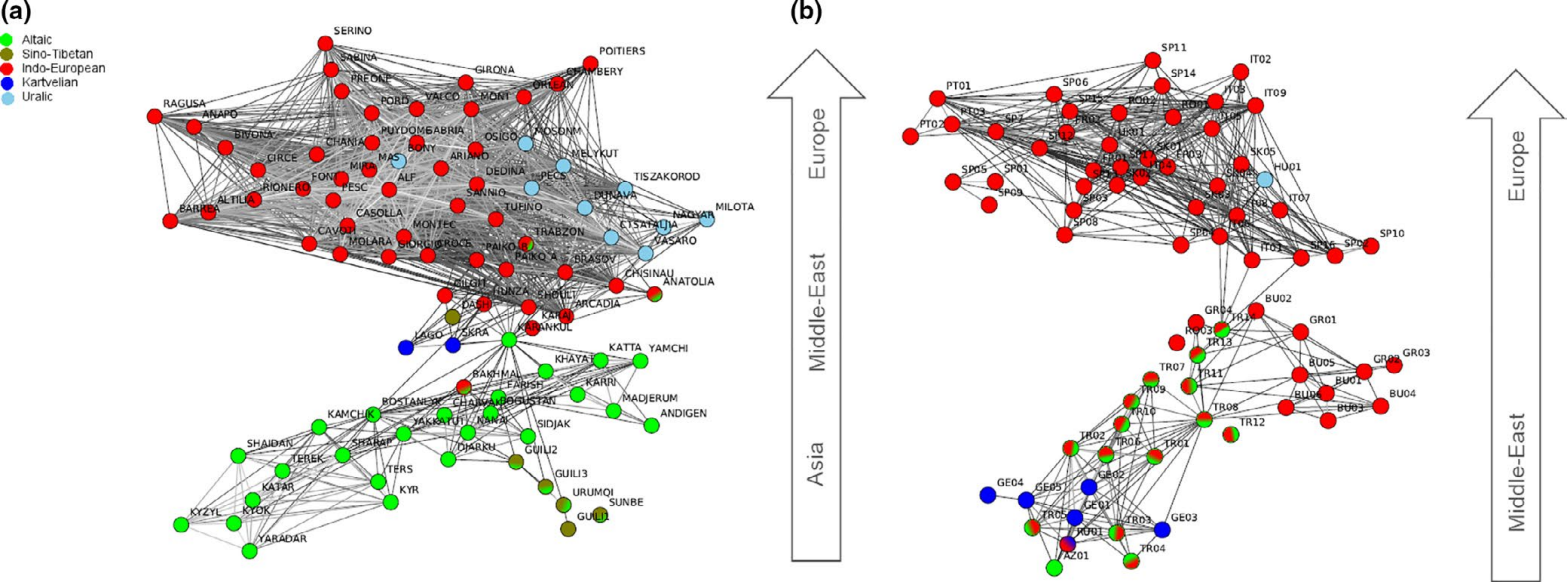
Graph network of 91 common walnut and 73 sweet chestnut populations in Eurasia. Nodes represent geographic sites and length of edges connecting nodes equivalent to genetic differentiation among the sites calculated using SSR markers for 91 common walnut (a) and 73 sweet chestnut (b) populations in Eurasia. The color of each node represents the language phylum spoken by human communities living in the geographic sampling sites

The hierarchical AMOVA revealed that the majority of the molecular variance was partitioned within *J. regia* (79.77%) and *C. sativa* (76.65%) individuals, while 11.75% and 7.93% was due to differences among language phylum areas, respectively (*p* < .0001). The remaining molecular variance, 8.49% for common walnut and 15.42% for sweet chestnut, was distributed among populations within language phylum groups. A multivariate population graph displayed a nonrandom pattern of association between the language phyla and the genetic differentiation inferred in common walnut and sweet chestnut populations across Eurasia (Figure 1). In particular, all tree populations located in sites where Indo‐European speakers were predominant (46 walnut populations: Pakistan, Tajikistan, Iran, Greece, Romania, Moldova, Slovakia, France, Spain, and Italy; 66 chestnut populations: Greece, Bulgaria, Romania, Slovakia, England, France, Spain, Portugal, and Italy) tended to cluster together. High genetic similarity was found between the majority of the tree populations growing in Central and Western Asian areas characterized by Altaic–Turkic speakers (31 walnut populations: Kyrgyzstan, Uzbekistan, Xinjiang‐Western China, and the chestnut population in Azerbaijan; Figure 1). Three distinct language phyla, Uralic, Kartvelian, and Sino‐Tibetan, are prevalent in three distinct walnut genetic subgroups, including Hungarian, Georgian, and Chinese germplasm, respectively. Congruent with walnut genetic distribution, five Georgian chestnut populations and the geographically proximal Russian site with Russian/Georgian speakers acted as a genetic bridge between the Middle East and Europe along with trees growing in Turkey where Turkic/Indo‐European bilingual speakers were located (Figure 1).

### 3.2 Word‐form richness and the genetic diversity of walnut and chestnut populations across Eurasia

We assigned a “word‐form richness” to each sampling site by collecting more than 70 and 52 word‐terms related to common walnut (Table S2) and sweet chestnut (Table S3), respectively, across Eurasia. We evaluated the level of tree genetic diversity by computing the allelic richness (*Rs*) parameter for each common walnut and sweet chestnut population based on SSR markers. By comparing these datasets, we detected statistically significant differences between genetic allelic richness (*Rs*) within *J. regia* and *C. sativa* populations, and the associated number of the related inherited terms, loanwords, and open compound words (Kruskal–Wallis tests: *p* < .05). The nonparametric post hoc Dunn's tests revealed that geographic sites with low *Rs* values were often associated with singletons (single word‐terms) in both species (Figure S3).

**Figure 2:**
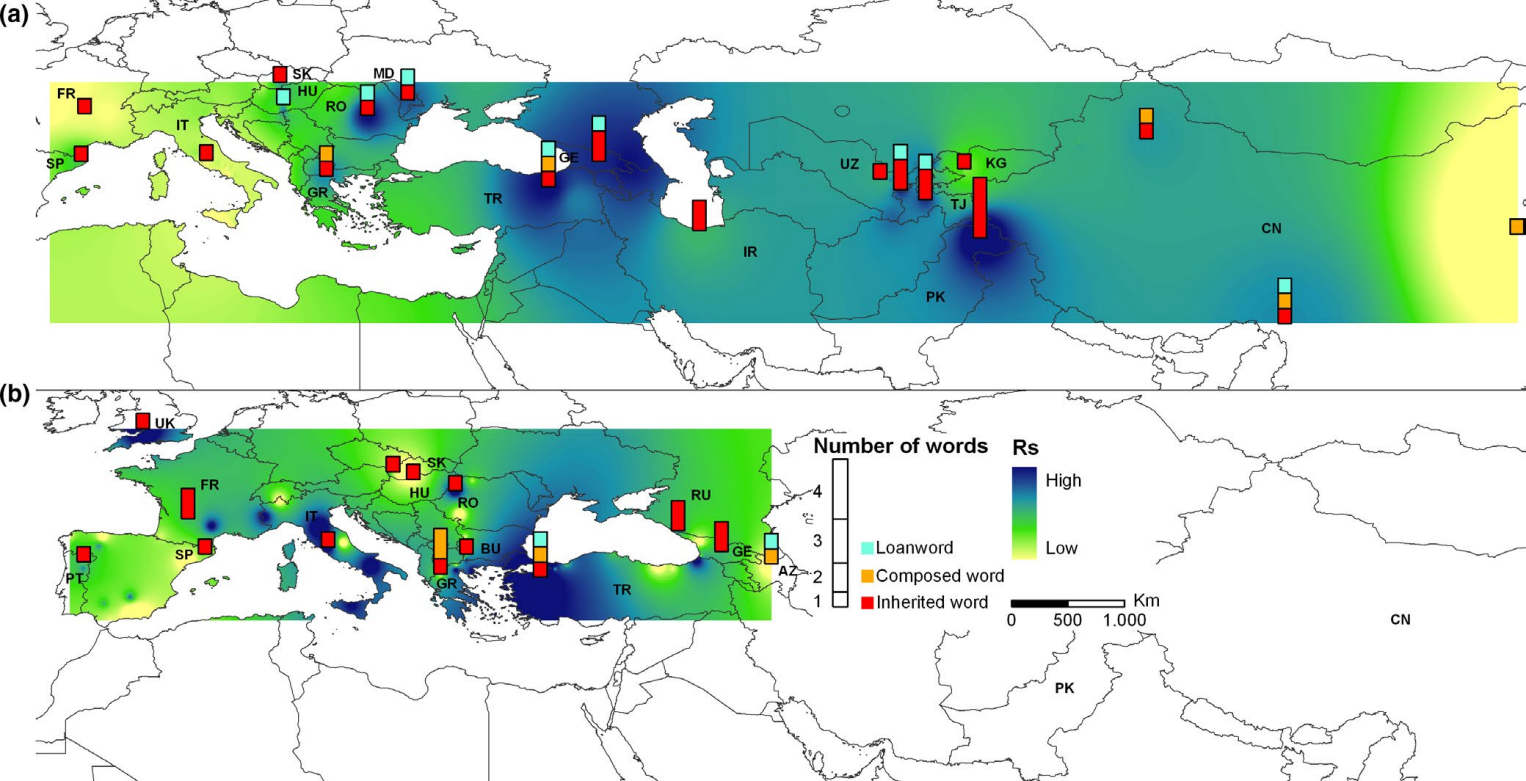
Spatial coincidences between genetic richness and stratification of walnut (a) and chestnut (b) linguistic terms. Hot spots of common walnut and sweet chestnut genetic diversity calculated using SSR markers and stratification of walnut and chestnut linguistic‐related forms across Eurasia. Inverse Distance Weighted (IDW) interpolations of the estimated allelic richness (Rs) values were modified from Pollegioni et al. (2017) and Mattioni et al. (2017)

**Figure 3:**
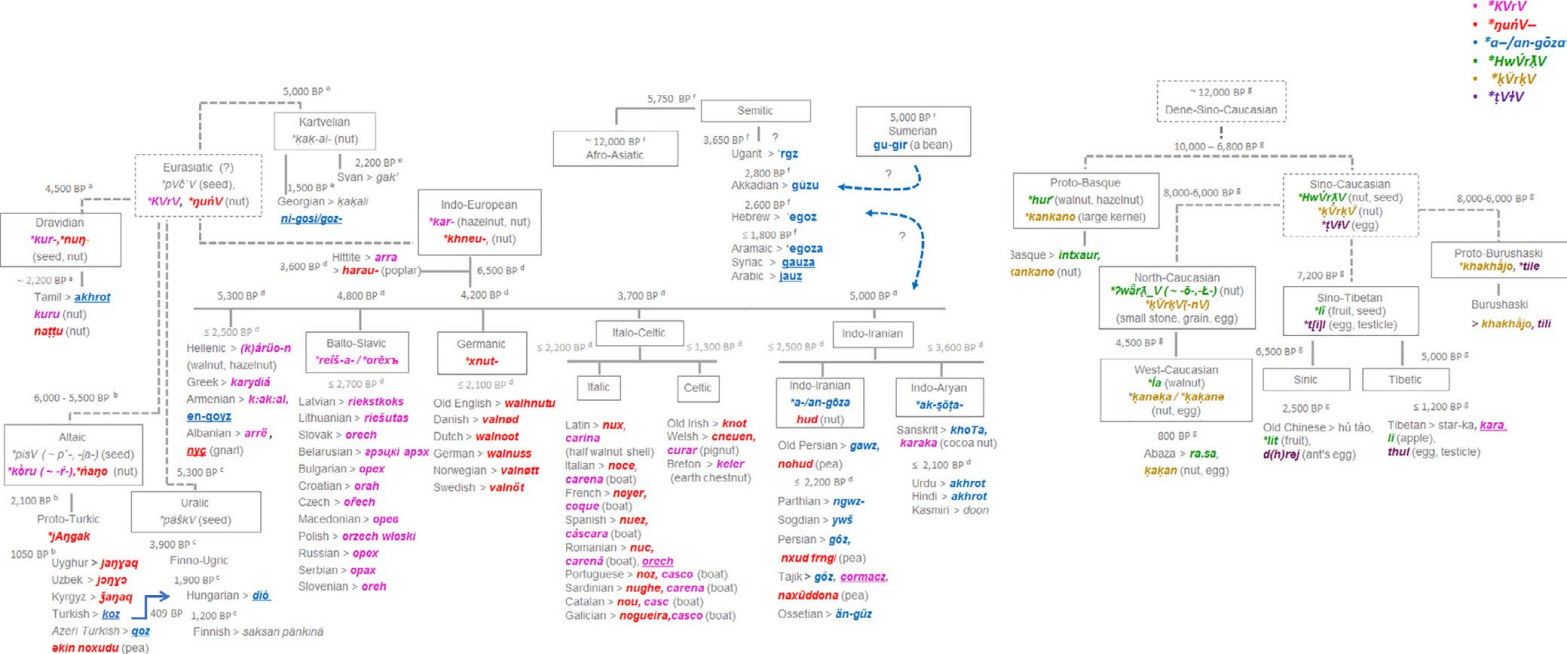
Linguistic evolution of the six major cognate sets (*KVrV, *ŋuńV‐, *a‐/an‐gōza, *HwV́rƛ̣V, *ḳV̆rḳV, *ṭVɫV) referred to walnut across Eurasia. The spatial and time distribution of walnut terms was derived using the consensus language trees with approximate estimation of the divergence times computed between languages of ^a^ Dravidian family (Kolipakam et al., 2018), ^b^ Altaic phylum‐Turkic family (Mikic et al., 2011; Savelyev & Robbeets, 2020), ^c^ Uralic phylum‐Finno‐Ugric family (Honkola et al., 2013), ^d^ Indo‐European phylum (Chang, Cathcart, Hall, & Garrett, 2015, in accordance with steppe theory), and ^e^ Kartvelian phylum (Koryakov, 2002) included into the putative Eurasiatic macro‐phylum as proposed by Pagel, Atkinson, Calude, and Meade (2013). ^f^ Afro‐asiatic phylum‐Semitic family (Kitchen et al., 2009), and ^g^ the putative Dene‐Sino‐Caucasian macro‐phylum (Van Driem, 2008) including Basque (Valdiosera et al., 2018), Burushaski, North‐Caucasian phylum (Koryakov, 2002), and Sino‐Tibetan phylum (Sagart et al., 2019) were also included in the reconstruction

The subsequent reconstruction of the synthetic interpolated map highlighted two macro‐areas, Central Asia and Trans‐Caucasus, with a spatial coincidence of high allelic richness and high number of word‐related inherited forms of walnut (Figure 2a). Gilgit‐Baltistan Province of Western‐Kashmir (Himalayas, Pakistan) showed a temporal stratification of four inherited terms, two in Urdu and Kashmiri Indo‐Aryan languages, *akhrot* (Indo‐Aryan proto‐word *ak‐ṣōṭa‐ ≤ 3,600 BP) and *doon,* and two in the ancient isolated Burushaski language from Dené‐Sino‐Caucasian super‐phylum, *khakhā́jo* and *tili* (Figure 3). The latter terms derived from the proto‐words *khakhā́jo and *tile dated back approximately to 8,000–6,000 BP. Burushaski walnut terms in the Hunza and Gilgit Valley of Pakistan was connected with phonetically similar words denoting egg and testicles in the Proto‐Sino‐Tibetan phylum (*t[i]l), large kernel in Proto‐Basque (*kankano) and small stone, grain and egg in Proto‐North Caucasian phylum (*ḳV̆rḳV̆(‐nV)). The adjacent Northern Pamir ridges of Tajikistan included one Persian walnut‐word *gôz* (Indo‐Iranian*a‐/an‐gōza ≤ 2,500 BP) and the loanword *cormacz* borrowed in the last century from Russian within the Indo‐European phylum (Figure 3). Similarly, Trans‐Caucasus sites in Georgia displayed a progressive overlay of three walnut‐word terms, the West‐Caucasian *ĺa dated back to 4,500 BP inherited from the Proto‐North Caucasian phylum *(ʔwǟrƛ̣_V ( ~ ‐ō‐,‐Ł‐)*, the Svan *gak’* (2,200 BP) or Georgian *ḳaḳali* (1,500 BP) belonging to Proto‐Karvelian *ḳaḳ‐al‐ (~5,000 BP), and *ni‐gosi/goz‐* borrowed into the Georgian from the Old Persian language. The ancient root *ʔwǟrƛ̣_V (~ ‐ō‐,‐Ł‐) served as the primary equivalent for the meaning “walnut” also in the Proto‐Basque (*hur̄), while, conversely, forms from the Proto‐Sino‐Tibetan word *HwV́rƛ̣V (nut, seed) with similar sound but different meaning were found in the Sinic (Old Chinese *lit = fruit) and Tibetic (Tibetan *li* = apple) groups (Figure 3). The proximal Anatolian sites included one extinct Proto‐Indo‐European (PIE) Hittite form *arra* (3,600 BP), the Turkic loanword *koz* (950–650 BP), and the current open compound word *ceviz ağacı*. It is worth mentioning that the eastern Himalayas of Western China showed high walnut genetic diversity and one autochthonous Sino‐Tibetan name of walnut *star‐ka*, and two loanwords, the Sino‐Tibetan Chinese *hú táo* (Silk Road theory, 2,250 BP) and the Indo‐European *kara* (≤1,200 BP) (Figures 2, 3, 2, 3; Table S2).

**Figure 4:**
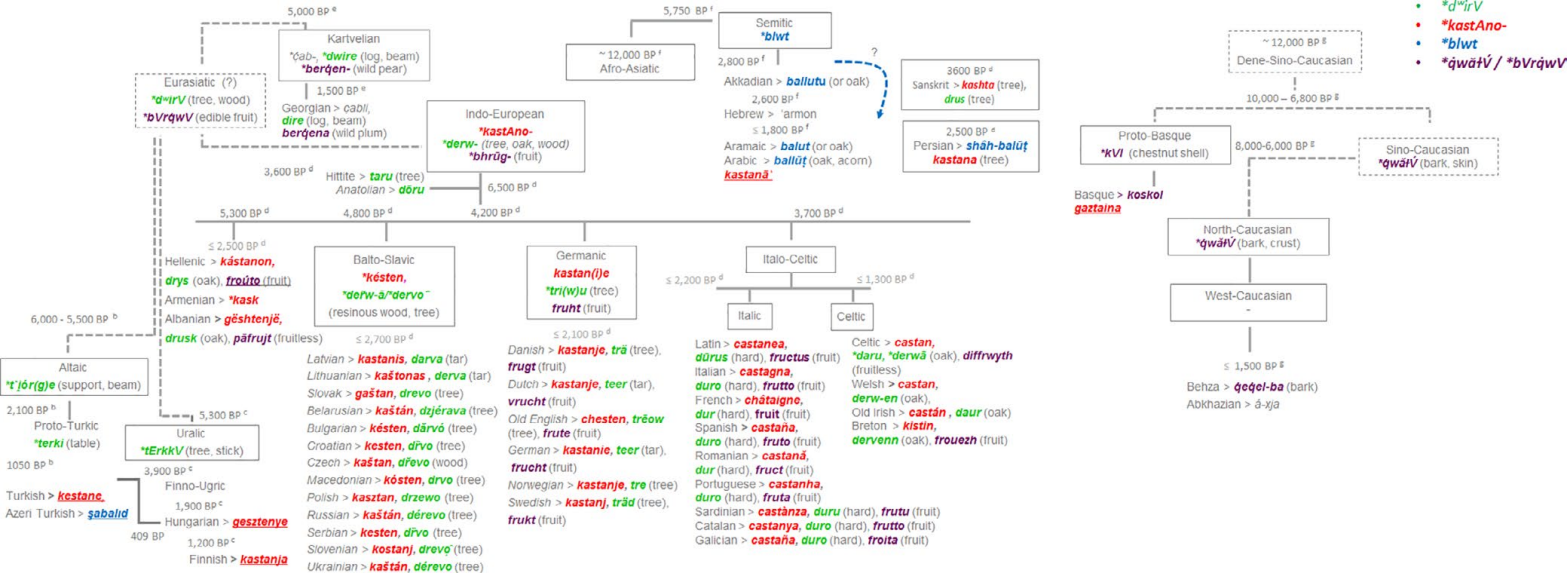
Linguistic evolution of the four major cognate sets (*kastAno‐, *derw‐, *blwt, *kVl) referred to chestnut across Eurasia. The spatial and time distribution of chestnut terms was derived using the consensus language trees with approximate estimation of the divergence times computed between languages of ^a^Dravidian family (Kolipakam et al., 2018), ^b^Altaic phylum‐Turkic family (Mikic et al., 2011; Savelyev & Robbeets, 2020), ^c^Uralic phylum‐Finno‐Ugric family (Honkola et al., 2013), ^d^Indo‐European phylum (Chang et al., 2015, in accordance with steppe theory), and ^e^Kartvelian phylum (Koryakov, 2002) included into the putative Eurasiatic macro‐phylum as proposed by Pagel et al. (2013). ^f^Afro‐asiatic phylum‐Semitic family (Kitchen et al., 2009), and ^g^the putative Dene‐Sino‐Caucasian macro‐phylum (Van Driem, 2008) including Basque (Valdiosera et al., 2018), North‐Caucasian phylum (Koryakov, 2002), and Sino‐Tibetan phylum (Sagart et al., 2019) were also included in the reconstruction

The co‐occurrence between the greater diversity of word forms and higher genetic diversity of *C. sativa* was detected in three macro‐regions of the species distribution: Caucasus‐Anatolian circuit, the Balkans, and Pyrenees Mountains (Figure 2b). Trans‐Caucasus sites in Georgia and Russia showed the autochthonous chestnut‐form of the North Caucasian language Abkhazian, *á‐xja* (≤ 1,500 BP), the Georgian *c̣abli* (≤ 1,500 BP) from the ancient proto‐Kartvelian *ć̣ab‐ (~5,000 BP), and the Russian *kaštán* within the Indo‐European phylum (≤2,700 BP) (Figure 4). Sites in Turkey included the Anatolian Indo‐European term *dōru* (3,600 BP), the Turkic loanword *kestane* (950–650 BP), and the current open compound word *bayat fıkra*. In the Balkans, Greek sampling sites shared with Bulgarian areas the Indo‐European inherited proto‐word *kastAno‐ (Hellenic *kástano* ≤ 2,500 BP), and displayed two open compound words *dios balanos* and *karua Euboikè*. Finally, the Aquitania site located in the Pyrénées Atlantiques (France) was characterized by a progressive accumulation of two chestnut terms. The Indo‐European French form *châtaigne* belonging from Latin *castanea* (≤2,200 BP) joined the Basque *koskol* from the Proto‐Basque relict form *kVl (chestnut shell, 8,000–6,000 BP). The etymology of *kVl belonged to a heterogenous core group ranging from the Proto‐Eurasiatic *bVrq̇wV (edible fruit) to Proto‐North‐Caucasian *q̇wăɫV* (bark, crust) (Figure 4).

### 3.3 Geographic coincidence between major cognitive sets and the genetic population structure of walnut and chestnut

**Figure 5:**
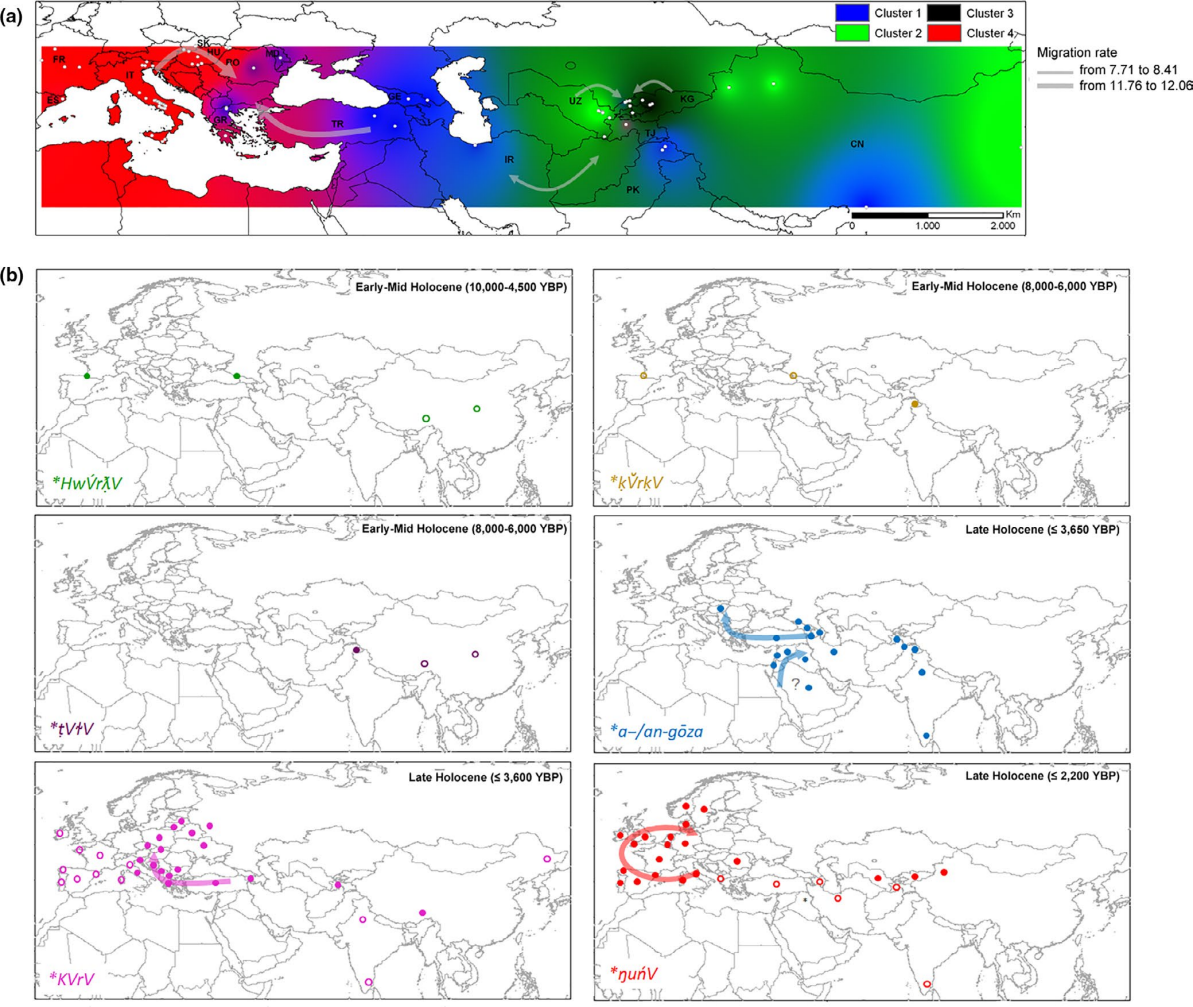
Geographic coincident between (a) the population genetic structure of walnut inferred across Eurasia and (b) the distribution of the six major cognate sets (*KVrV, *ŋuńV‐, *a‐/an‐gōza, *HwV́rƛ̣V, *ḳV̆rḳV, *ṭVɫV)) referred to walnut. Inverse Distance Weighted (IDW) interpolations of the estimated mean population membership values (cluster analysis: Qi) were modified from Pollegioni et al. (2017). Gray arrows indicated migration rates per generation. Word forms connected to proto‐words but changing meaning over the time are reported as empty dots. The earliest date of appearance of the attested walnut linguistic terms was provided for each cognitive set

**Figure 6:**
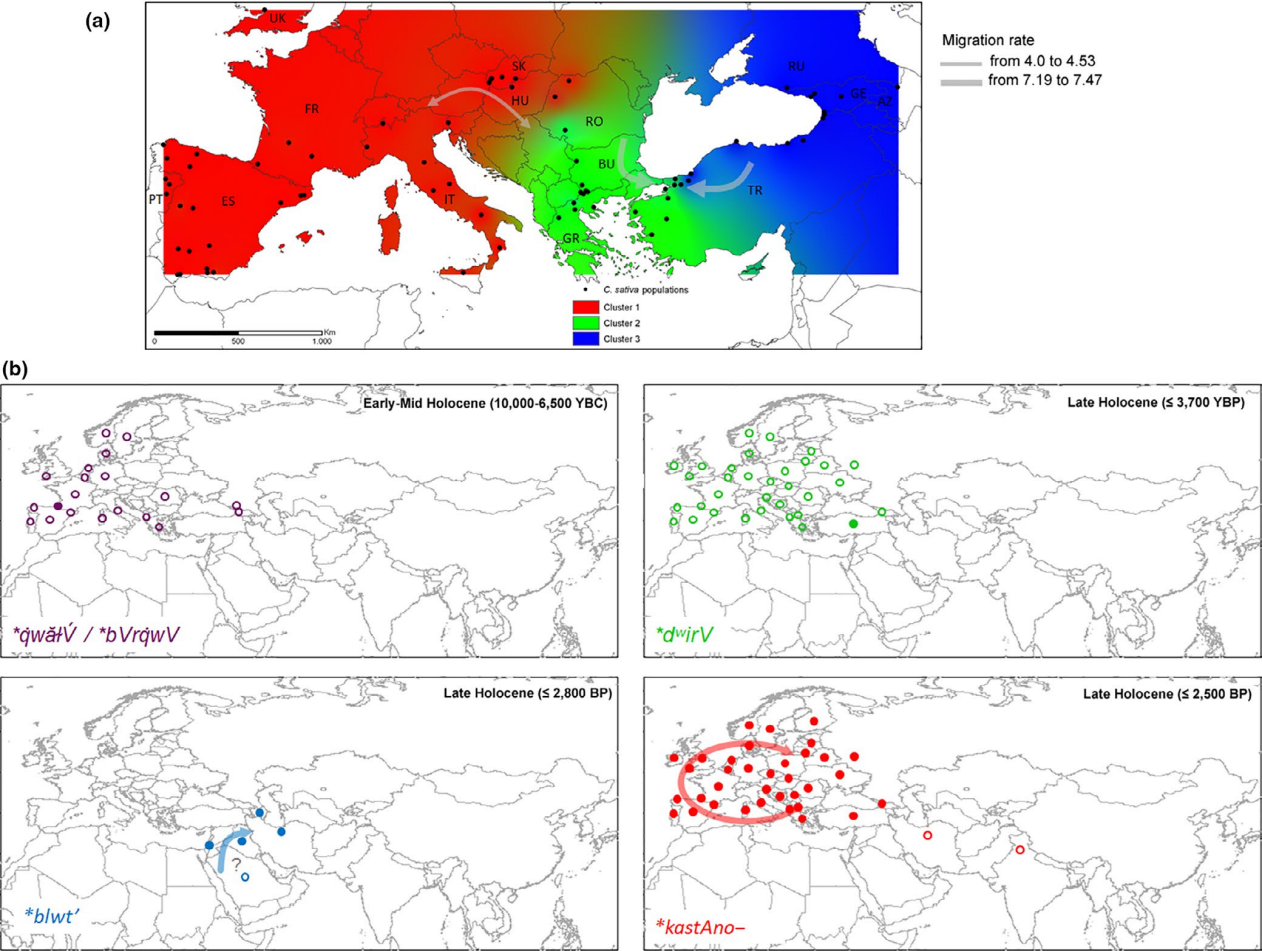
Geographic coincident between (a) the population genetic structure of chestnut inferred across Eurasia and (b) the distribution of the four major cognate sets (*derw‐, *kastAno‐, *blwt, *kVl) referred to chestnut. Inverse Distance Weighted (IDW) interpolations of the estimated mean population membership values (cluster analysis: Qi) were modified from Mattioni et al. (2017). Gray arrows indicated migration rates per generation. Word forms connected to proto‐words but changing meaning over the time are reported as empty dots. The earliest date of appearance of the attested chestnut linguistic terms was provided for each cognitive set

A geographic congruence was detected between the geographic distribution of walnut/ chestnut‐language major cognitive sets and the spatial genetic structure of *J. regia* and *C. sativa* populations across Eurasia. STRUCTURE analysis of the 91 common walnut and 73 sweet chestnut populations based on SSR markers indicated four (*K*_walnut_ = 4) and three (*K*_chestnut_ = 3) as the most appropriate number of genetic clusters, respectively (Figures 5, 6). The number of migrants per generation was also estimated for all possible pairs of the inferred clusters in *J. regia* and *C. sativa* populations. Between 8.41 and 1.65 migrants per generation have been exchanged between walnut clusters, except for six Balkan populations admixed between cluster 1 and cluster 4, showing the highest levels of migration ranging from 11.76 (Balkans vs. Western‐Central Asia) to 12.06 (Balkans vs. Western Europe) migrants per generation (Figure 5). Between 2.31 and 4.53 migrants per generation were exchanged between chestnut clusters located in Eurasia, except for three Central‐Turkish populations admixed between cluster 2 and cluster 3, showing the highest levels of migration ranging from 7.19 (Central Turkey vs. Balkans + Western Turkey) to 7.47 (Central Turkey vs. Eastern Turkey + Trans‐Caucasian) migrants per generation (Figure 6). Six major cognate sets referred to walnut terms, *HwV́rƛ̣V, *ḳV̆rḳV, *ṭVɫV, *a‐/an‐gōza, *ŋuńV‐, and *KVrV (Table 2) and four major cognate sets referred to chestnut terms*q̇wăɫV́, *blwt, *dʷirV, and *kastAno‐ (Table 3) were identified across Eurasia. Statistical differences in the distribution of all major cognitive sets were detected among the inferred genetic clusters of common walnut and sweet chestnut populations (Table 4).

**Table 2:** The six major cognate sets (*HwV́rƛ̣V, *ḳV̆rḳV, *ṭVɫV, *a‐/an‐gōza, *KVrV, *ŋuńV‐) referred to walnut terms across Eurasia

Phylum	Word forms for walnut			
	Family	Language	Proto‐word/word form	Meaning
Sino‐Dene Caucasian *HwV́rƛ̣V (nut, seed)				
Proto‐Basque			*hur̄	walnut, hazelnut
		Basque	*intxaur*	walnut
Sino‐Tibetan			*lĭ	fruit, seed
		Old Chinese	*‘lit*	fruit
		Tibetan	*li*	apple
North Caucasian			*ʔwǟrƛ̣_V ( ~ ‐ō‐,‐Ł‐)	nut
	West‐Caucasian		*ĺa	walnut
		Abaza	*ra.sa*	walnut
Sino‐Dene Caucasian *ḳV̆rḳV̆́ (nut)				
Proto‐Basque			*kankano	large kerner
		Basque	*kankano*	nut
Proto‐Burushaski			*khakhā́jo	walnut
		Burushaski	*khakhā́jo*	walnut
North Caucasian			*ḳV̆rḳV̆(‐nV)	small stone, grain, egg
	West‐Caucasian		*ḳanǝḳa/ *ḳaḳanǝ	nut, egg
		Abaza	*ḳaḳan*	nut, egg
Sino‐Dene Caucasian *ṭVɫV (egg)				
Proto‐Burushaski			*tile	walnut
		Burushaski	*tili*	walnut
Sino‐Tibetan			*t[i]l	egg, testicle
		Old Chinese	*d(h)rǝj*	ant's egg
		Tibetan	*thul*	egg, testicle
Indo‐European or Afro‐Asiatic (*a‐/an‐gōza)				
Indo‐European			*‐*	‐
	Indo‐Iranian		*a‐/an‐gōza	something hidden inside a shell
		Old Persian	*gawz*	walnut
		Parthian	*ngwz‐*	walnut
		Sogdian	*ywš*	walnut
		Persian	*gôz*	walnut
		Tajik	*gôz*	walnut
		Ossetian	*än‐gũz*	walnut
	Indo‐Aryan		*ak‐ṣōṭa*‐	walnut
		Sanskrit	*AkhoTa*	walnut
		Urdu	*akhrot*	walnut
		Hindi	*akhrot*	walnut
‐	‐	Sumerian (?)	*gu‐gir*	a bean
Semitic			‐	‐
		Akkadian	*gūzu*	walnut
		Ugaritic	*'rgz*	walnut
		Hebrew	*‘egoz*	walnut
		Aramaic	*‘egoza*	walnut
		Arabic	*jauz* ^a^	walnut
		Syriac	*gauza* ^a^	walnut
Altaic			*‐*	‐
		Turkish	*koz* ^a^	walnut
		Azeri Turkish	*qoz* ^a^	walnut
Uralic			*‐*	‐
		Hungarian	*dió* ^a^	walnut
Eurasiatic *KVrV (nut)				
Altaic			*kŏ̀ru ( ~ ‐ŕ‐)	nut
Dravidian			*kur‐	seed, nut
		Tamil	*kuru*	nut
Indo‐European			*kar‐	nut, hazelnut
		Hittite	*arra*	walnut
	Hellenic		*(k)árüo‐n*	walnut, hazelnut
		Greek	*karydiá*	walnut
	Proto‐Armenian		*k:ak:al*	walnut
		Armenian	*k'ak'al*	walnut
	Proto‐Albanian		*arrë*	walnut
		Albanian	*arrë*	walnut
	Balto‐Slavic		*reîš‐a‐/*orēxъ	walnut
		Latvian	*riekstkoks*	walnut
		Lithuanian	*graikinis riešutas*	walnut
		Slovak	*orech*	walnut
		Belarusian	*гpэцкi apэx*	walnut
		Bulgarian	*opex*	walnut
		Croatian	*orah*	walnut
		Czech	*ořech*	walnut
		Macedonian	*opeв*	walnut
		Polish	*orzech włoski*	walnut
		Russian	*opex*	walnut
		Serbian	*opax*	walnut
		Slovenian	*oreh*	walnut
		Ukrainian	*volos'kyy horikh*	walnut
	Celtic		*‐*	‐
		Old Irish	*curar*	pignut
		Breton	*keler*	earth chestnut
	Italic		‐	‐
		Latin	*carina*	boat, half walnut shell
		Italian	*carena*	boat
		French	*coque*	boat
		Spanish	*cáscara*	boat
		Romanian	*carenă*	boat
		Portuguese	*casco*	boat
		Sardinian	*carena*	boat
		Catalan	*casc*	boat
		Galician	*casco*	boat
	Indo‐Aryan		‐	‐
		Sanskrit	*karaka*	cocoa nut shell
	Indo‐Iranian	‐	‐	‐
		Tajik	*cormacz* ^a^	walnut
Sino‐Tibetan	Tibetic		*‐*	‐
		Tibetan	*kara* ^a^	walnut
Eurasiatic *ŋuńV (nut)				
Dravidian			*nuŋ‐	nut
		Tamil	*naṭṭu*	nut
Altaic			*ńaŋo	nut
	Turkic		*jAŋgak	walnut
		Kyrgyz	*ǯaŋaq or ǯaŋɣaq*	walnut
		Northern Uzbek	*jɔŋɣɔq*	walnut
		Uyghur	*jaŋɣaq*	walnut
		Azeri Turkish	*əkin noxudu*	pea
Indo‐European			*khneu‐	nut
		Hittite	*harau‐*	poplar
		Albanian	*nyç*	gnarl
	Germanic		*xnut‐	nut
		Danish	*valnød*	walnut
		Dutch	*walnoot*	walnut
		Old English	*walhnutu*	walnut
		German	*walnuss*	walnut
		Norwegian	*valnøtt*	walnut
		Swedish	*valnöt*	walnut
	Celtic	‐	*‐*	‐
		Welsh	*cneuen*	walnut
		Old Irish	*knot*	walnut
	Italic	‐	*‐*	‐
		Latin	*nux*	walnut
		Italian	*noce*	walnut
		French	*noyer*	walnut
		Spanish	*nuez*	walnut
		Romanian	*nuc*	walnut
		Portuguese	*noz*	walnut
		Sardinian	*nughe*	walnut
		Catalan	*nou*	walnut
		Galician	*nogueira*	walnut
	Indo‐Iranian			
		Old Persian	*nohud*	pea
		Modern Persian	*nxud frngi*	pea
		Tajik	*naxūddona*	pea

^a^ Word loan from different language phylum.

**Table 3:** The four major cognate sets (*q̇wăɫV́, *blwt, *dʷirV, *kastAno‐) referred to chestnut term across Eurasia

Phylum	Word forms for chestnut			
	Family	Language	Proto‐word/word form	Meaning
Eurasiatic *bVrq̇wV (edible fruit) and Sino‐Caucasian *q̇wăɫV́ (bark, skin)				
Proto‐Basque			*kVl	chestnut shell
		Basque	*koskol*	chestnut
North Caucasian			*q̇wăɫV	bark, crust
	West‐Caucasian		*‐*	
		Behza	*q̇eq̇el‐ba*	bark
Kartvelian			*berq̇en‐	wild pear
		Georgian	*berq̇ena*	wild plum
Indo‐European			*bhrūg‐	fruit
		Greek	*froúto* ^a^	fruit
		Albanian	*pāfrujt*	fruitless
	Germanic		*fruht*	fruit
		Danish	*frugt*	fruit
		Dutch	*vrucht*	fruit
		Old English	*frute*	fruit
		German	*frucht*	fruit
		Norwegian	*frukt*	fruit
		Swedish	*frukt*	fruit
	Celtic		*diffrwyth*	fruitless
		Breton	*frouezh*	fruit
	Italic		‐	‐
		Latin	*fructus*	fruit
		Italian	*frutto*	fruit
		French	*fruit*	fruit
		Spanish	*fruto*	fruit
		Romanian	*fruct*	fruit
		Portuguese	*fruta*	fruit
		Sardinian	*frutu*	fruit
		Catalan	*frutto*	fruit
		Galician	*froita*	fruit
Indo‐European or Afro‐Asiatic *blwt' (?)				
Indo‐European			*‐*	‐
	Indo‐Iranian		*‐*	‐
		Old Persian	*šāh‐balūṭ*	chestnut
Semitic			‐	‐
		Akkadian	*ballutu*	chestnut/oak
		Aramaic	*balut*	chestnut/oak
		Arabic	*ballūṭ*	oak, acorn
Altaic			*‐*	‐
		Azeri Turkish	*şabalıd* ^a^	chestnut
Eurasiatic *dʷirV (tree, wood)				
Altaic			*t`i̯ór(g)e	support, beam
	Turkic		*terki	table
Uralic			*tErkkV	tree, stick
Kartvelian			*dwire	log, beam
		Georgian	*dire*	log, beam
Indo‐European				
	Proto‐Anatolian		*tṓru‐	wood, tree
		Hittite	*taru*	tree
		Anatolian	*dōru*	chestnut
	Hellenic		*drũs*	oak
		Greek	*drys*	oak
	Proto‐Albanian		*dru*	wood, log
		Albanian	*drusk*	oak
	Baltic		*der̃w‐ā̂	resinous wood
		Latvian	*darva*	tar
		Lithuanian	*derva*	tar
	Slavic		*dervo	tree
		Slovak	*drevo*	tree
		Belarusian	*dzjérava*	tree
		Bulgarian	*dǎrvó*	tree
		Croatian	*dȑvo*	tree
		Czech	*dřevo*	wood
		Macedonian	*drvo*	tree
		Polish	*drzewo*	tree
		Russian	*dérevo*	tree
		Serbian	*dȑvo*	tree
		Slovenian	*drevọ̑*	tree
		Ukrainian	*kdérevo*	tree
	Germanic		*tri(w)u	tree
		Danish	*trä*	tree
		Dutch	*teer*	tar
		Old English	*trēow*	tree
		German	*teer*	tar
		Norwegian	*tre*	tree
		Swedish	*träd*	tree
	Celtic		*daru, *derwā	oak
		Welsh	*derw‐en*	oak
		Old Irish	*daur*	oak
		Breton	*dervenn*	oak
	Italic		‐	‐
		Latin	*dūrus*	hard
		Italian	*duro*	hard
		French	*dur*	hard
		Spanish	*duro*	hard
		Romanian	*dur*	hard
		Portuguese	*duro*	hard
		Sardinian	*duru*	hard
		Catalan	*duro*	hard
		Galician	*duro*	hard
Indo‐European *kastAno‐				
Altaic				
	Turkic		‐	‐
		Turkish	*kestane* ^a^	chestnut
Indo‐European				
	Hellenic		*kástanon*	chestnut
		Greek	*kástano*	chestnut
		Albanian	*gështenjë*	chestnut
	Balto‐Slavic		*késten	chestnut
		Latvian	*kastanis*	chestnut
		Lithuanian	*kaštonas*	chestnut
		Slovak	*gaštan*	chestnut
		Belarusian	*kaštán*	chestnut
		Bulgarian	*késten*	chestnut
		Croatian	*kesten*	chestnut
		Czech	*kaštan*	chestnut
		Macedonian	*kósten*	chestnut
		Polish	*kasztan*	chestnut
		Russian	*kaštán*	chestnut
		Serbian	*kesten*	chestnut
		Slovenian	*kostanj*	chestnut
	Germanic		*kastan(i)e*	chestnut
		Danish	*kastanje*	chestnut
		Dutch	*kastanje*	chestnut
		Old English	*chesten*	chestnut
		German	*kastanie*	chestnut
		Norwegian	*kastanje*	chestnut
		Swedish	*kastanj*	chestnut
	Celtic		*castan*	chestnut
		Welsh	*castan*	chestnut
		Old Irish	*castán*	chestnut
		Breton	*kistin*	chestnut
	Italic		‐	‐
		Latin	*castanea*	chestnut
		Italian	*castagna*	chestnut
		French	*châtaigne*	chestnut
		Spanish	*castaña*	chestnut
		Romanian	*castană*	chestnut
		Portuguese	*castanha*	chestnut
		Sardinian	*castànza*	chestnut
		Catalan	*castanya*	chestnut
		Galician	*castaña*	chestnut
	Indo‐Iranian		‐	‐
		Sanskrit	*kashta*	tree
		Persian	*kastana*	tree
Uralic				
	Finno‐Ugric		‐	‐
		Finnish	*kastanja* ^a^	chestnut
		Hungarian	*gesztenye* ^a^	chestnut

^a^ Word loan from different language phylum.

**Table 4:** Distribution of the major cognitive sets referred to walnut term (*HwV́rƛ̣V, *ḳV̆rḳV, *ṭVɫV, *a‐/an‐gōza, *KVrV, *ŋuńV‐) and chestnut term (*q̇wăɫV́, *blwt, *dʷirV, *kastAno‐) across the genetic clusters of walnut and chestnut populations inferred by SSR markers

**Walnut cognitive sets**						
**Walnut Clusters** ^a^	*HwV́rƛ̣V	*ḳV̆rḳV	*ṭVɫV	*a‐/an‐gōza	*KVrV	*ŋuńV‐
1 (*n* = 10)	2 (20%)	2 (20%)	2 (20%)	9 (90%)	4 (40%)	1 (10%)
2 (*n* = 12)	0	0	0	0	0	11 (91.7%)
3 (*n* = 10)	0	0	0	0	0	10 (100%)
Admixed 2 × 3 (*n* = 8)	0	0	0	1 (12.5%)	1 (12.5%)	8 (100%)
4 (*n* = 45)	0	0	0	10 (22.2%)	1 (2.22%)	34 (75.6%)
Admixed 1 × 4 (*n* = 10)	0	0	0	0	6 (100%)	0
χ^2^ (*df* = 5)	42.3	42.3	42.3	26.4	36.9	25.2
*P*	< 0.001	< 0.001	< 0.001	< 0.001	< 0.001	< 0.001
**Chestnut cognitive sets**						
**Chestnut Clusters** ^a^		*q̇wăɫV́	*blwt	*dʷirV	*kastAno‐	
1 (*n* = 47)		2 (4.26%)	0	0	47 (100%)	
2 (*n* = 17)		0	0	4 (23.6%)	17 (100%)	
3 (*n* = 17)		0	1 (5.89%)	7 (41.2%)	11 (64.7%)	
Admixed 2 × 3 (*n* = 3)		0	0	3 (100%)	3 (100%)	
χ^2^ (*df* = 3)		40.2	42.1	27.7	30.1	
*p*		<.001	<.001	<.001	<.001	

Note

The number of populations (*n*) included in each genetic cluster was reported in brackets. Statistically significant difference in distribution (*Chi‐squared* test) was reported for each cognate set.

^a^ The expected numbers of populations are computed under the hypothesis of priori probability equal to 0.5.

In *J. regia*, three small ancient cognate classes of the Dene‐Sino‐Caucasian super‐phylum, *HwV́rƛ̣V, *ḳV̆rḳV, and *ṭVɫV, were scattered only in few common walnut populations (20%) of the inferred cluster 1 (Table 4), two populations in the Georgian sites for *HwV́rƛ̣V and Hunza and Gilgit Valley sites of Pakistan for *ḳV̆rḳV, and *ṭVɫV (Figure 5, Table 4). The distribution of nine reflexes of the PIE form *a‐/an‐gōza was concentrated in western and south‐central Asia, including all common walnuts of cluster 1 sampled from four Trans‐Caucasus sites (Georgia, Turkey), Iran, Kashmir‐western Himalayas (Pakistan), and Tibet‐eastern Himalayas, except one population (Karankul) in Uzbekistan (Table 4, Figure 5). The *a‐/an‐gōza cognate set was attested across a number of unrelated language families, spreading in particular across a range of Semitic languages from the extinct Ugarit (*‘rgz,* 3,650 BP) and Akkadian (*gūzu*, 2,800 BP) to current Aramaic (*egoz*), Syriac (*gauza*), and Arabic (*jauz*) (Figure 3, Figure 5). The proto‐Altaic form for walnut *ńaŋo, borrowed from the Proto‐Euro‐Asiatic etymologic root *ŋuńV, was recognized in many modern Turkic languages including Kyrgyz (*ǯaŋaq or ǯaŋɣaq*), Northern Uzbek (*jɔŋɣɔq*), and Uyghur (*jaŋɣaq*). The Central‐Asian distribution of *ńaŋo term overlapped with the inferred genetic clusters 2 and 3 and admixed populations between cluster 2 and 3, including all common walnut populations from, Kyrgyzstan, northern China, and Uzbekistan. Apart from ten populations sampled in Hungary in which the loanword *dió* was borrowed from the Turkic language (409 BP) and Crete (Greece), the remaining 35 European common walnut populations included in the genetic cluster 4 (Spain, Italy, France, Slovakia) displayed a co‐dispersal with the related Euro‐Asiatic *ŋuńV > PIE *khneu, mainly restricted to Italic (*xnut), Celtic (*knūs*), and Germanic languages (*xnut) (Latin *nux*, ≤2,200 BP). Finally, the inherited terms from the PIE *kar borrowed from the Proto‐Euro‐Asiatic *KVrV were co‐distributed with the five easternmost Balkan populations showing admixed profiles between cluster 1 and cluster 4. Although its geographic distribution was wide across all Indo‐European languages, the proto‐form *kar referred to the common walnut in the Anatolian‐Hittite (*arra*, 3,600 BP), Old Hellenic (*(k)árüo‐n,* (≤ 2,500 BP), Latin (*caria*, ≤ 2,200 BP), Albanian (*arrë*), Greek (*karydiá*), and in all Balto‐Slavic languages with *reîš‐a‐ and *orēxъ as a Baltic and Slavic proto‐form (Figure 3, Figure 5).

In *C. sativa*, the populations from eastern Europe were separated in two main groups (Figure 4); cluster 1 included populations from Russia, Azerbaijan, Georgia, and eastern Turkey, whereas the populations from Romania, Bulgaria, Greece, and western Turkey were grouped in cluster 2 according to STRUCTURE analysis. The Portuguese, Spanish, Italian, Slovakian, and Hungarian and two sweet chestnut populations from Romania were included in cluster 3. Despite this sharp genetic spatial repartition of the 73 sweet chestnut populations into three main clusters, the Indo‐European cognate *kastAno‐ was predominant across Europe and Western Asia (Table 4, Figure 6). This etymon was preserved with its original sense in the Proto‐Armenian *kask, Old Hellenic *kástanon* (≤2,500 BP), Albanian *gështenjë,* Proto‐Balto‐Slavic *késten, Germanic *kastan(i)e*, Celtic *castan,* and Latin *castanea* (≤2,200 BP). The overlay approach suggested *kastAno‐term penetrance in the Hungarian language via Turkic‐elite dominance scenario (*gesztenye,* 409 BP) and through South‐Western Asia in the Turkish (*kestane*) and Arabic (*kastanāʾ*) territories. A small ancient cognate class of the Dene‐Sino‐Caucasian super‐phylum, *q̇wăɫV́, was restricted to two Basque chestnut populations of Western Europe included in cluster 3 (Table 4, Figure 6). The basic meaning of the PIE form *derw‐ “tree,” derived from the Proto‐Euro‐Asiatic etymologic root *dʷirV (tree, wood), evolved in “tree” and/or “tar” in the Anatolian‐Hittite (*taru*), Germanic (*tri(w)u) and Balto‐Slavic languages (*der̃w‐ā̂, *dervo), “wood” in Albanian (*dru*), “hard” in the Italic (*dūrus*), “oak” in Old Hellenic (*drũs*), Celtic (*daru, *derwā) but also “chestnut” in the ancient Anatolian languages (*dōru*) (3,600 BP). A fourth cognite set *blwt’ was detected in the Levant and Iran with a semantic shift from oak to chestnut and *vice versa* (Figures 4, 6).

## 4 DISCUSSION

### 4.1 Genetic boundaries among tree populations and languages diversity in human communities

In this study, spatial analysis showed that the inferred barriers to gene flow among tree populations coincided with large differences in human language. Genetic differentiation computed between common walnut and sweet chestnut populations and the divergence in current human language phylogeny were both positively correlated with geographic distances between sampled sites. Language and genetic tree data are therefore not statistically independent, exhibiting significant spatial autocorrelation. As postulated by Gavin et al. (2013), biological and linguistic diversity respond in similar ways to drivers and constraints such as environmental and topographic variables. Gorenflo, Romaine, Mittermeier, and Walker‐Painemilla (2012) noted a strong correlation between linguistic diversity and habitat heterogeneity, as measured by more‐diverse habitats and higher topographic complexity. Similarly, the observed separation of J. regia and C. sativa populations reflected, at least in part, a complex topography of habitats—the presence of high mountains, deep valleys, and deserts—that prevented pollen/seed dispersal during the Holocene in Eurasia (Mattioni et al., 2017; Pollegioni et al., 2017). Our data are consistent with this theory, since the topographic boundaries affecting patterns of diversity in common walnut and sweet chestnut plants also influenced the geographic patterns of language phyla (for more details see the next section “Co‐occurrence of word‐term richness and genetic diversity of walnut and chestnut across Eurasia”).

Topography is not the sole determinant of spatial patterns of diversity, however; a large body of research characterizes human migrations and socio‐cultural factors such as political complexity and social connectedness as additive drivers behind the diversification of languages or their geographic homogenization over the time (Currie & Mace, 2009; Gavin et al., 2013). The association between ancient language phyla and the genetic structure of tree populations remained significant (albeit low) even after adjustment for geographic distances. Similarities in human language over large geographic areas might have facilitated the dispersal of common walnut and sweet chestnut, their introduction to new habitats, and the genetic homogenization of disparate populations. These data are consistent with the existence of long‐distance connections and ancient historical factors promoting human‐mediated seed exchanges within culturally similar language macro‐areas across Eurasia starting from ~2,500 BP onward (Persian, Greek and Roman civilizations, for more details see the next section “ Tracing indirect and direct human‐mediated dispersal routes of walnut and chestnut across Eurasia”). An interdependence between material and cultural exchange‐drive components was clearly illustrated in crop species such as cassava (Delêtre et al., 2011), maize (Orozco‐Ramírez et al., 2016), pearl millet (Naino Jika et al., 2017), and sorghum (Labeyrie et al., 2014). While the cultural boundaries limited the connectivity of human communities and impeded the diffusion of annual plant material among different ethnolinguistic groups, historical–cultural similarities strongly promoted exchanges of seeds and knowledge by facilitating social relationships within members of the same macro‐groups.

There are numerous obvious differences between common walnut and sweet chestnut trees and annual crops (e.g., cereals or legumes) for which parallel demic and cultural expansion is assumed across Eurasia (Fort, 2012). Small seed size, long storage life, short life cycle, and easy cultivation contributed to the adoption of many annual crops. They provided immediate sources of food in large quantities on relatively small areas. These traits promoted the domestication and long‐distance dispersal of annual crops throughout the continents from the Early Neolithic (Bar‐Yosef, 2017). In sharp contrast, common walnut and sweet chestnut trees are very demanding in terms of soil quality and sensitive to temperature oscillations. Both species produce suborthodox and temperate‐recalcitrant seeds, whose nursing is difficult (Bonner, 2008), but both species are relatively precocious, bearing seeds in less than nine years. Adult trees can remain productive for at least 40 years for common walnut and 100 years for sweet chestnut, and their nuts can survive up to two years under simple storage conditions (Bonner, 2008; Vahdati et al., 2019). Thus, short‐ and long‐distance transportation of these seeds mediated by humans cannot be excluded from our scenario. As reported by Bar‐Yosef (2017), during the second half of the Holocene people transported seeds, animals, and technologies across Eurasia within few months by rivers or sea and by land with support of draft animals (e.g., donkeys, horses, and camels). Considering all those elements and the wide use of common walnut and sweet chestnut as religious, spiritual, and ritual plants (e.g., seeds exchanged as inherited via gifts at weddings as symbol of fertility), food, and medicine, it is reasonable to evoke the homophily theory as a plausible explanation for part of the large‐scale genetic patterns of these two species in Eurasia. Similar to small‐scale farming systems (Pautasso et al., 2013), seed exchanges might have occurred between members of the same historical macro‐groups as a result of their frequent and preferential interactions (Leclerc & Coppens d’Eeckenbrugge, 2012). We cannot rule out that these types of seed transactions as a component of human cultural institutions, as well as typical economic transactions such as barter and the simple act of carrying familiar, convenient food on a journey, shaped the genetic structure and diversity of J. regia and C. sativa across Eurasia. Over time, humans and their crops coevolved in response to climate changes, socio‐economic pressures, and the cultural significance of crops and seeds. The importance of these human interactions in the spread of annual crops seems almost intuitive, but it is much less so for tree species that remained undomesticated, even if they were widely cultivated.

### 4.2 Co‐occurrence of word‐term richness and genetic diversity of walnut and chestnut across Eurasia

Our analysis revealed higher diversity in terminology was associated with higher allelic richness (*Rs*) in samples from (a) the Himalayas and Pamir Ridge of Central‐South Asia (for common walnut), (b) Pyrenees Mountains of Western Europe (for sweet chestnut), and (c) Trans‐Caucasus and Anatolia‐Balkan circuit for (both species) in the Eastern Mediterranean. The Himalayas, Trans‐Caucasus, and Pyrenees Mountains are considered glacial refugia and reservoirs of high levels of genetic diversity for many plant species, including common walnut and sweet chestnut (Médail & Diadema, 2009). Local foci of high genetic diversity occurred in these Eurasian refugia where *J. regia* and *C. sativa* survived after Pleistocene glaciations. The earliest traces of *Juglans regia*‐type fossil pollen were deposited in Central Asia during the Upper Pleistocene (12,000–40,000 years BP) and the species persisted through the beginning of the Holocene (10,000–12,000 years BP) in the Himalayas, in the western Kunlun mountains of the Tibetan Plateau, in the Pamir mountains of southern Tajikistan and in the Tien Shan mountains of Northeast China (Pollegioni et al., 2015). Chestnuts, as well as walnuts, survived cold climatic eras as fragmented populations confined in micro‐refugia of the Italian and Iberian Peninsulas. It is well recognized that the so‐called Colchis refugia (i.e., Southern Caucasus, Western Georgia) and the Turkish‐Balkan regions offered the best conditions for the survival of several thermophilous plants including *C. sativa* and *J. regia* (Krebs et al., 2019). In the same macro‐regions, marked concentration of localized idiosyncratic terms related to walnut and chestnut were detected, mainly reflecting the enormous phylogenetic diversity in languages recorded in such geographic areas (Gavin et al., 2013).

At the global scale, ecoregions of high biological diversity often coincide with hotspots of indigenous‐linguistic diversity (Gorenflo et al., 2012). Studies in biogeography suggested that environmental and spatial heterogeneity as well as socio‐cultural factors affected biological processes and language evolution/persistence in every continent (Amano et al., 2014). Vicariance appears to act similarly on human and plant populations, generating inter‐ and intrademe diversification. High levels of topographic complexity may have generated barriers to the movement of people, increased the potential for geographic isolation, and promoted the divergence of ethnolinguistic groups as a result of drift and adaptation to complex and fragmented environmental habits (Gavin et al., 2013). Similarly, we postulate that the heterogeneous environments of Himalayas, Trans‐Caucasus and Pyrenees Mountains promoted common walnut and sweet chestnut diversity as well as the evolution and/or persistence over thousands of years of languages in different language families spoken by a small number of people in isolated enclaves. At least one additional historical process may contribute to local peaks in diversity; the Himalayas and Trans‐Caucasia are classified as “accretion zones,” where many language macro‐families are present and the structural diversity of languages is high and increases over time through immigration (Nichols, 1997). The availability of a large set of walnut and chestnut related terms in such biodiversity hotspots gave us the unique opportunity to confirm long‐term persistence and use of common walnut and sweet chestnut in the cultures of these regions during the second half of the Holocene. The terminological diversity detected in the Basque Country, Trans‐Caucasus, and Kashmir Valley partially reflected a progressive temporal stratification of word‐terms related to *J. regia* and *C. sativa* passing from Dené‐Sino‐Caucasian (~8,000–6,000 BP), Kartvelian (~5,000 BP) to Indo‐European (~≤ 5,300 BP) word‐terms. Similarly, both species have been closely associated with human activities in these primary centers of fruit tree diversity since the Neolithic or Early Bronze Age onward.

In particular, *J. regia* nuts have been found as usual component of ordinary meals in Ceramic‐Neolithic culture (4,650–3,950 BP) at Kanispur and Burzahom sites of Kashmir Valley, (Lone, Khan, & Buth, 1993; Pokharia, Mani, Spate, Betts, & Srivastava, 2018), where two ancient Burushaski (*khakhā́jo* and *tili*), and three Indo‐European (Kashmiri *doon*, Urdu *akhrot*, and Old Persian *gawz*, ≤3,600 BP) walnut word‐terms were highlighted. There is evidence of the religious importance of common walnut in Kashmir from the 1st millennium BC onward; walnut became the essential element of several rituals in the Shaivism religion of Kashmir (Tikoo, 2013, ~2,800 BP) and pre‐Buddist Bön religion in the Tibetan area of Eastern Himalayas (Weckerle, Huber, Yongping, & Weibang, 2005; ~2,515 BP).

Although the human consumption of common walnut and sweet chestnut seeds has been demonstrated in Aceramic (9,450–8,450 BP) and Ceramic (8,450–7,450 BP) Neolithic cultures of Central Turkey (Siddiq, 2016), both species seems to be connected with religious beliefs, cults and rituals only from the EBA in the Balkan‐Colchis circuit. Concurrently with North‐Caucasus Majkop culture (5,850–4,850 BP), recognized as the putative homeland of the Northwestern Caucasian language family (walnut proto‐term *ĺa, Wang et al., 2019), a wide variety of plant foods including, grape (*Vitis vinifera*), hazelnut (*Corylus*), common walnut, and sweet chestnut were stored in ceramic vessels as offerings for votive/ritual purpose in the geographically adjacent settlements of the Kura‐Araxes culture in the Southern Caucasus (Areni‐1 Cave, Armenia 6,230–5,790 BP, Wilkinson et al., 2012; Aradetis Orgora, Georgia, 4,950 BP, Kvavadze et al., 2019). While it is still matter of debate if Kartvelian was the idiom spoken by Kura‐Axes communities (Beridze, 2019, walnut proto‐term *ḳaḳ‐al‐, chestnut proto‐term *ć̣ab‐), the subsequent Indo‐European speakers of the Early Kurgan settlements in Georgia showed similar habits using animals, cereals, hazelnuts, figs (*Ficus carica* L.), walnuts (Indo‐European proto‐form *kar and *a‐/an‐gōza), and chestnuts (Indo‐European proto‐form *kastAno‐ and *blwt') as major components of funeral gifts (Bedeni Plateau, 4,450–4,150 BP, Kvavadze et al., 2015; Ananauri‐3‐kurgan site, 4,450 BP, Makharadze, 2015). All these studies suggested that common walnut and sweet chestnut cultivation, together with viticulture and pasturing, was fully incorporated in the Trans‐Caucasian agricultural landscape of EBA. These early horticultural practices were substantially expanded to Late Bronze–Early Iron Age (Namcheduri hillock, 3,650–3,550 BP, Giorgadze & Inaishvili, 2015), and Early Middle Age Trans‐Caucasia (Tsitsamuri site, 1,650–1,450 BP, Kvavadze, Rukhadze, Nikolaishvili, & Mumladze, 2008).

Finally, charcoal fragments of *C. sativa* were uncovered in the Neolithic site of Kobeaga II cave in the Spanish Basque Country (7,780 BP, Roces‐Díaza, Jiménez‐Alfarod, Chytrýf, Díaz‐Varelag, & Álvarez‐Álvarezc, 2018) and Aquitaine region of French Basque Country (Krebs et al., 2019). Although introduction of common walnut and sweet chestnut to France and Spanish was typically associated with “Roman globalization,” the presence of both species was detected during the emergence of proto‐historic urban centers in Central France (Corent Plateau, 3,640–3,340 BP, Ledger, Miras, Poux, & Milcent, 2015) and in Spanish Menorca Island (Cova de Cárritx, 3,400–2,750 BP, Stika, 1999) during Late Bronze Age (LBE). In these cases, archeological evidence supports the idea of early local cultivation of both species in LBE communities of France and Spain, maybe reflecting the early establishment of trade with people in adjacent Mediterranean regions where walnut and chestnut were native, including Basque Country (Peña‐Chocarro & Zapata Peña, 2003).

### 4.3 Tracing indirect and direct human‐mediated dispersal routes of walnut and chestnut across Eurasia

In this study, we detected a partial spatial congruence between the distribution of language terms and their proto‐words with the inferred genetic clusters of common walnut and sweet chestnut populations. The great variety of modern forms can be described in terms of six major cognate sets referred to walnut terms, *HwV́rƛ̣V, *ḳV̆rḳV, *ṭVɫV, *a‐/an‐gōza, *ŋuńV‐, and *KVrV and four major cognate sets referred to chestnut terms *q̇wăɫV́, *blwt, *dʷirV, and *kastAno‐, with distinctive distributions for both species.

Large cognate class sizes with high congruence with genetic clusters were observed for walnut cognates *a‐/an‐gōza, *ŋuńV‐, and *KVrV, and for chestnut cognates, *blwt, and *kastAno‐. As formulated by Gamkrelidze and Ivanov (1995), the spread of the descendants of Eurasian *KVrV > Indo‐European *kar walnut‐proto‐form westwards from Anatolia to Balkans is mirrored by the genetic hybridization in the Balkans sites between cluster 1 (Anatolia) and cluster 4 (Western Europe) walnut germplasm. The genetic modeling analysis of 91 walnut populations suggested that walnut genotypes from Anatolia hybridized with autochthonous Balkan trees in southeastern Europe over several generations starting from the EBA (5,630–4,140 BP) (Pollegioni et al., 2017). Similarly, chestnut populations near the Dardanelle strait were mixtures of two gene pools detected in western Turkey (cluster 2) and Eastern Turkey–Trans‐Caucasus region (cluster 3), with a high migration rate per generation. It is recognized that the Late Neolithic or the Chalcolithic coincided with abrupt increments in pollen fossil deposits of common walnut and sweet chestnut in eastern Mediterranean and the Italian–Spanish peninsulas (Krebs et al., 2019; Pollegioni et al., 2017). During the Holocene Thermal Maximum period (11,000–5,000 BP), the circum‐Mediterranean lands along with those areas of Southwestern Asia experienced warm and humid climate (Renssen, Seppä, Crosta, Goosse, & Roche, 2012). An increase of the human impact associated with forest clearing by fire for agriculture was also recorded in the second half of the Holocene, resulting in a gradual increase in arable and grazing land (Roberts, Brayshaw, Kuzucuoglu, Perez, & Sadori, 2011). Partially promoted by these favorable climatic conditions, complex societies developed across Eurasia, particularly in the eastern Mediterranean Basin during the Bronze Age. The EBA cultures prospered and stimulated the exchanges of ideas and new technologies including wheel‐made pottery, metallurgy, and an advanced form of agriculture in the Aegean‐Anatolian‐Caucasus circuit. Therefore, as proposed by Krebs et al. (2019), we concur that the admixed Balkan genotypes represent the spontaneous expansion of common walnut and sweet chestnut during the mid‐Holocene starting from neighboring Eurasian refugia, fostered by warm‐humid climate and indirect human influence (large‐scale woodland clearances). It is noteworthy that genetic data for trees and the dispersal routes of the Indo‐European *kar proto‐form coincided astonishingly well with the complex history of human migration as recently reconstructed by whole‐genome sequencing analysis of ancient DNA (Mathieson et al., 2018). After farming spread from Anatolia westward along a Mediterranean route and northwestward via the Danubian route in the mid‐seventh millennium BC, southeastern Europe continued to be a bridge between east and west macro‐areas with human gene admixture of Balkan regions with Anatolian sites throughout the Neolithic, Chalcolithic and Bronze Age (6,000–4,000 BP), underscoring the relative permeability of the territories to forest ecosystems and humans. Considering the cultural significance of fruit trees in EBA societies, it is plausible to assume human preferential exploitation, selection, and preservation of common walnut and sweet chestnut trees in patches of remaining woodland in Eastern Mediterranean.

It is also true that we cannot exclude the adoption of *kar among cultures that had the most successful engagement in inter‐regional exchanges of seeds across the Near East and the Aegean region from the 6th millennium BP onward. However, we did not find similar congruence with chestnut genetic data and any Indo‐European word proto‐form. The recovery of plant remains of fig, grape and olive (*Olea europaea*) along with seeds of non‐native fruit species such as pine (*Pinus pinea*) and pomegranate (*Punica granata*) from the Late Bronze Age shipwreck at Ulu Burun, Turkey (3,350 BP), revealed the existence of a complex, sophisticated maritime trade network dominated by the proto‐Phoenicians more than three millennia ago (Haldane, 1993; Ward, 2003). The archeological evidence indicated the extensive exchange of luxury goods for elite consumption with elaborate symbolic meanings in the Middle and Late Bronze Age Eastern Mediterranean cultures. Nevertheless, the scarcity of common walnut and sweet chestnut from the Bronze Age maritime archeo‐botanical remains may suggest that people did not regularly trade these nuts across the ancient Eastern Mediterranean until the Hellenistic and Roman period, as proved by the detection of DNA sequences of *Juglans regia*‐type in 2,400‐ to 2,200‐year‐old jars recovered from Greek shipwrecks (Foley, Hansson, Kourkoumelis, & Theodoulou, 2012) and by the presence of assorted amphoras filled with common walnuts, figs, olives, wine, oil, and *garum* (aromatic fish sauce) in several Roman vessels (e.g., Marausa, Italy, 1,800 BP, https://www.livescience.com/19901‐smuggled‐cargo‐ancient‐roman‐shipwrecl.html).

Surprisingly, there were no reports of sweet chestnut remains from any Mediterranean shipwreck excavations until the Carolingian Middle Age (Jarman, Hazell, Campbell, Webb, & Chambers, 2019). Nevertheless, the predominant denomination of the sweet chestnut with the Latinized form of Greek *kástanon* and Armenian *kast* (*castanea*) and the subsequent wide spread of the Indo‐European proto‐form *kastAno‐ attested the growing importance of nondomesticated chestnut for fruit, fodder and timber production during the Greek and Roman Empire. Even so, sweet chestnut was mainly consumed by the low social classes and was subsidiary to common walnut (Allevato, Saracino, Fici, & Di Pasquale, 2016). Similarly, the second Indo‐European proto‐form for walnut *khneu‐, from the Euro‐Asiatic proto‐form *ŋuńV, progressively replaced the original derived terms of *kar in the Italic‐Celtic‐Germanic languages. According to Gamkrelidze and Ivanov (1995), *khneu‐ inherited words initially did not refer to *J. regia* through its non‐native range in Central Europe but to the nut tree or hazelnut which was common in the Early Holocene. In connection with Roman campaigns, *khneu‐ derived terms were translated as “walnut”, attesting its key role in the Roman agro‐forest management across Europe (Allevato et al., 2016; Bakels & Jacomet, 2003). In the German languages, the term “*walnut*” (OE *welh‐hnutu*) in fact derived from a compound meaning, *wealh* = foreign + hnutu − nut. The genetic homogeneity of sweet chestnut (cluster 3) and common walnut (cluster 4) populations inferred across Western and part of Eastern Europe confirmed the main expansion of these species throughout Europe occurred after 2,500 BP, as a result of Roman fruit cultivation and their commercial activities (Peña‐Chocarro & Zapata Peña, 2003).

Finally, our integrated analysis detected a spatial coincidence between the genetic clustering of walnut populations sampled from Western and Central Asia (cluster 1) and the geographic dispersal of the third cognate set of common walnut, the Old Persian proto‐word *a‐/an‐gōza. As proposed by Pollegioni et al. (2015), walnut management and the Old Persian term *gôz* co‐dispersed through long‐distance trade across the Persian Empire starting from the Achaemenid phase (2,450–2,280 BP). Although the language root of *a‐/an‐gōza* has been classified as Indo‐Iranian, its origin is uncertain. In the Afro‐Asiatic phylum, this cognate set is present as loanwords from the Persian language such as Syriac *gauza* and Arabic *jauz* but also as inherited terms from unknown Afro‐Asiatic proto‐form in three extinct Semitic languages, the Aramaic *egoz* (2,850–3,850 BP), the Mesopotamian Akkadian *gūzu* (~ 4,350 BP), and Levantin Ugaritic *'rgz* (3,400–4,400 BP) (Kitchen, Ehret, Assefa, & Mulligan, 2009). Similarly, the third cognate set of chestnut *blwt refers to chestnut in the Old Persian language (*šāh‐balūṭ*) and chestnut/ oaks in Aramaic (*balut*), Akkadian (*ballutu*), and Assyrian (*ballutuu*). The adoption of non‐Indo‐European proto‐words for common walnut and sweet chestnut by Iranian tribes cannot be excluded. Recently, cuneiform scripts provided tangible evidence of Mesopotamian‐Assyrian royal gardens and buildings for ex situ conservation of fruit trees such as almond, date, olive, pomegranate, fig, grapevine pear, and walnut (Bowe, 2015). In particular, the Nimrud Royal Garden of Neo‐Assyrian King Ashurnasirpal II (2,833–2,809 BP) and the Nineveh Hanging Gardens of King Sennacherib (2,655–2,631 BP), erroneously identified as the Hanging Gardens of Babylon, boasted of walnut groves and oaks. Considering all this information, and the refugium probability patterns computed by Krebs et al. (2019) in Eurasia, the putative prominent role of the Near Est as a secondary refugium and source population for later wide dispersal of walnut and chestnut species deserves further exploration.

## 5 CONCLUSION

In this study, we detected a partial geographic congruence between ethno‐linguistic repartition of human communities, the distribution of major cognitive sets, and the inferred genetic clusters of common walnut and sweet chestnut populations across Eurasia. Our data indicated that IBD processes, landscape heterogeneity, and cultural boundaries might have promoted both human language diversification and walnut/chestnut differentiation across the same geographic macro‐regions. In particular, we found three hotspots of common walnut and sweet chestnut genetic diversity associated to high linguistic‐related form richness: Himalayas, Trans‐Caucasus, and Pyrenees Mountains. In agreement with genetic and linguistic data, the archeological evidence suggested a long‐term interaction between walnuts, chestnuts, and people living in these macro‐areas. An early integration of common walnut and sweet chestnut cultivation in the Trans‐Caucasian agricultural landscape of EBA cultures was postulated. Our multidisciplinary approach supported the indirect and direct role of humans in shaping the walnut and chestnut diversity across Eurasia, in particular during Persian Empire and Greek–Roman colonization, until the first evidence of active selection and clonal propagation by grafting of both species.

We are aware that our reconstruction of walnut and chestnut history will be affected by adjustments and revisions based on the availability of linguistic databases and theoretical developments in linguistics. As explained by Moran, Grossman, and Verkerk (2020), variability in phonological inventories, development of new phylogenetic tools, redefinition of cultural/linguistic lineages, and dating methods progressively increase the knowledge of the major processes underlying the evolution of human language. Despite these limitations, our findings highlight the importance and cogency (*i*) an efficient integration of the relevant cultural factors in the classical genome (G) × environmental (E) model, and (*ii*) a systematic application of the biocultural diversity concept in the reconstruction of evolutionary history of tree species.

## 6 CONFLICT OF INTEREST STATEMENT

The authors declare no conflicts of interest.

## AUTHOR CONTRIBUTION

**Paola Pollegioni:** Conceptualization (lead); Data curation (lead); Formal analysis (lead); Funding acquisition (equal); Investigation (equal); Methodology (lead); Project administration (equal); Supervision (equal); Validation (lead); Visualization (equal); Writing‐original draft (lead); Writing‐review & editing (lead). **Stefano Del Lungo:** Conceptualization (equal); Data curation (supporting); Investigation (supporting); Methodology (equal); Supervision (equal); Validation (supporting); Writing‐review & editing (supporting). **Ruth Müller:** Conceptualization (supporting); Formal analysis (supporting); Project administration (supporting); Supervision (supporting); Writing‐original draft (supporting); Writing‐review & editing (supporting). **Keith Woeste:** Conceptualization (supporting); Funding acquisition (supporting); Methodology (supporting); Project administration (supporting); Supervision (equal); Writing‐review & editing (supporting). **Francesca Chiocchini:** Data curation (supporting); Methodology (supporting); Software (supporting); Visualization (equal); Writing‐review & editing (supporting). **Jo Clark:** Project administration (supporting); Resources (supporting); Validation (supporting); Writing‐review & editing (supporting). **Gabriel Hemery:** Project administration (supporting); Resources (supporting); Validation (supporting); Writing‐review & editing (supporting). **Sergio Mapelli:** Project administration (supporting); Resources (supporting); Validation (supporting); Writing‐review & editing (supporting). **Fiorella Villani:** Conceptualization (supporting); Formal analysis (supporting); Resources (supporting); Validation (supporting); Writing‐review & editing (supporting). **Maria Emilia Malvolti:** Conceptualization (equal); Data curation (supporting); Funding acquisition (supporting); Project administration (supporting); Resources (supporting); Supervision (supporting); Validation (supporting); Writing‐review & editing (supporting). **Claudia Mattioni:** Conceptualization (equal); Data curation (equal); Formal analysis (supporting); Investigation (equal); Resources (supporting); Supervision (equal); Validation (equal); Writing‐original draft (equal); Writing‐review & editing (equal).

## Supporting information

Supplementary Material

## Data Availability

The SSR dataset for common walnut populations is available from the figshare repository (https://doi:10.6084/m9.figshare.4665235.v1) and for sweet chestnut populations from the TreeGenes Database, accession number TGDR068.
