# Supplementary material for: Biocultural diversity of common walnut (Juglans regia L.) and sweet chestnut (Castanea sativa Mill.) across Eurasia
Source: Ecol Evol. 2020 Sep 24;10(20):11192–216. doi: 10.1002/ece3.6761 (PMC7593191; doi:10.1002/ece3.6761)
Supplement: Supplementary file 1 — Supplementary Material [file ECE3-10-11192-s001.pdf]

Supplemental Information_1 for:

Biocultural diversity of *Juglans regia* (L.) and *Castanea sativa* (Mill.) across Eurasia

Paola Pollegioni, Keith E. Woeste, Stefano Del Lungo, Francesca Chiocchini, Jo Clark, Gabriel E. Hemery, Sergio Mapelli, Ruth Müller, Fiorella Villani, Maria Emilia Malvolti, Claudia Mattioni

Table of Contents:

**Fig. S1**	Page 2
**Fig. S2**	Page 3
**Fig. S3**	Page 4
**Reference Figures**	Page 5
**Table S1**	Page 6
**Table S2**	Page 13
**Table S3**	Page 18
**References Table**	Page 22

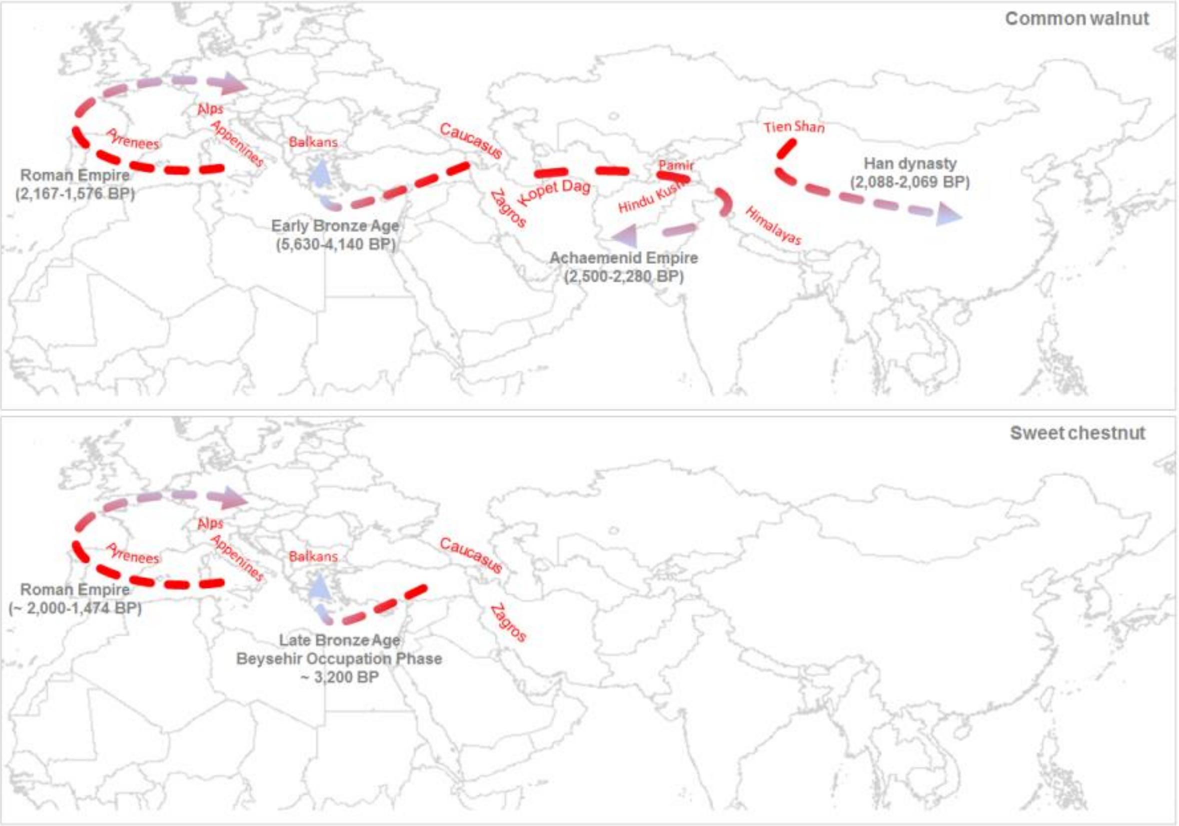

Figure S1. Major historical dispersal events of common walnut and sweet chesnut across their native range, as postulated by Pollegioni et al., (2017), Mattioni et al., (2017) and Krebs Pezzatti, Beffa, Tinner & Conedera (2019) based on genetic patterns inferred using SSR neutral markers and fossil pollen analysis. Location of putative mountains-glacial refugia of both species was reported in red.

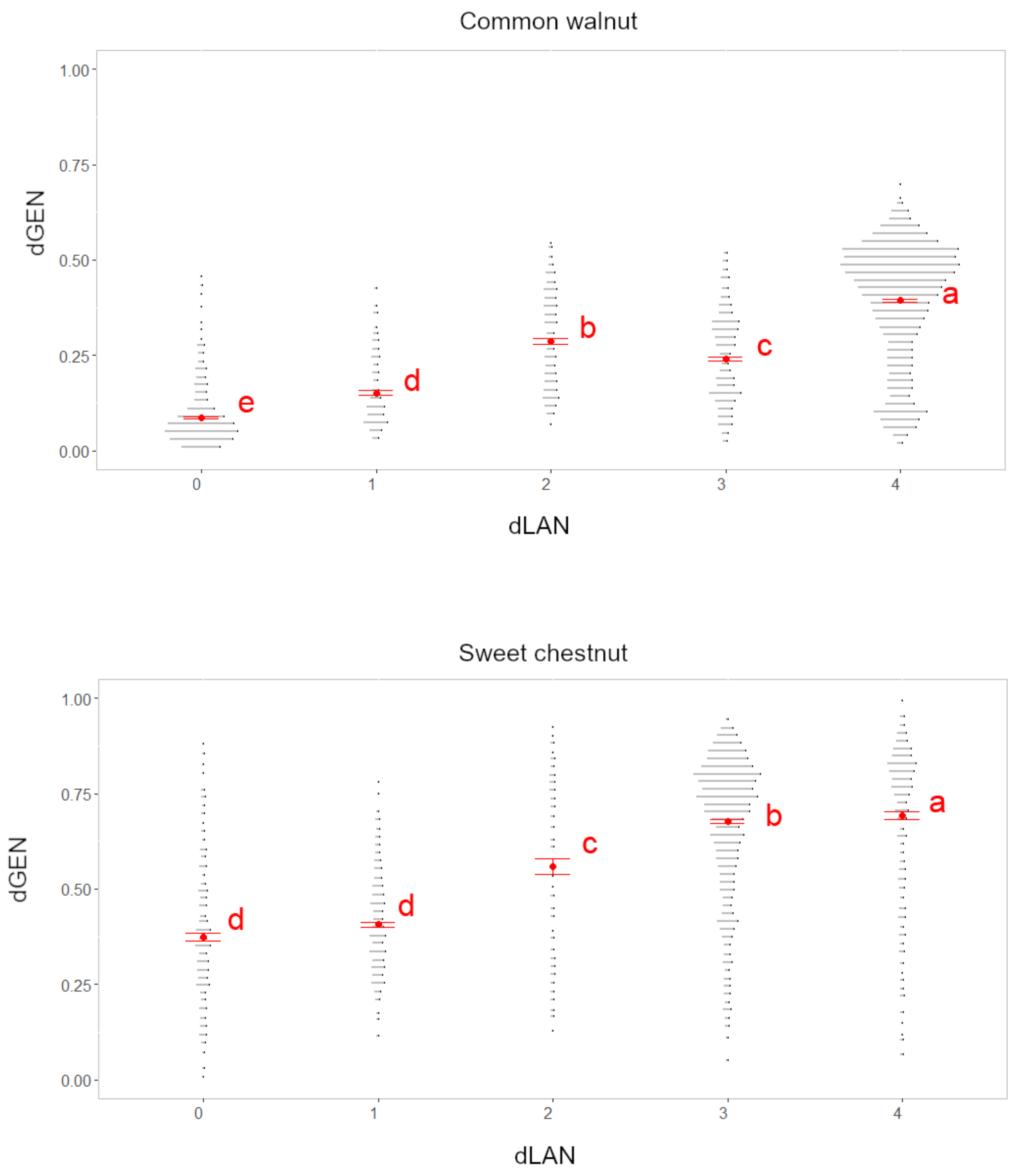

Figure S2. Dot plots comparing tree genetic distances (d_{GEN}) computed using Jost, (2008) coefficient and human linguistic distances (d_{LAN}) calculated on the basis of The Ethnologue website' s classification of languages (Gordon, 2005) among 91 walnut geographic sites and 73 chestnut geographic sites. Mean (red dot) and standard error (red bar) are reported. Mean values showing the same letter were not significantly different at $P \geq 0.05$ according to the non-parametric post hoc Dunn's test.

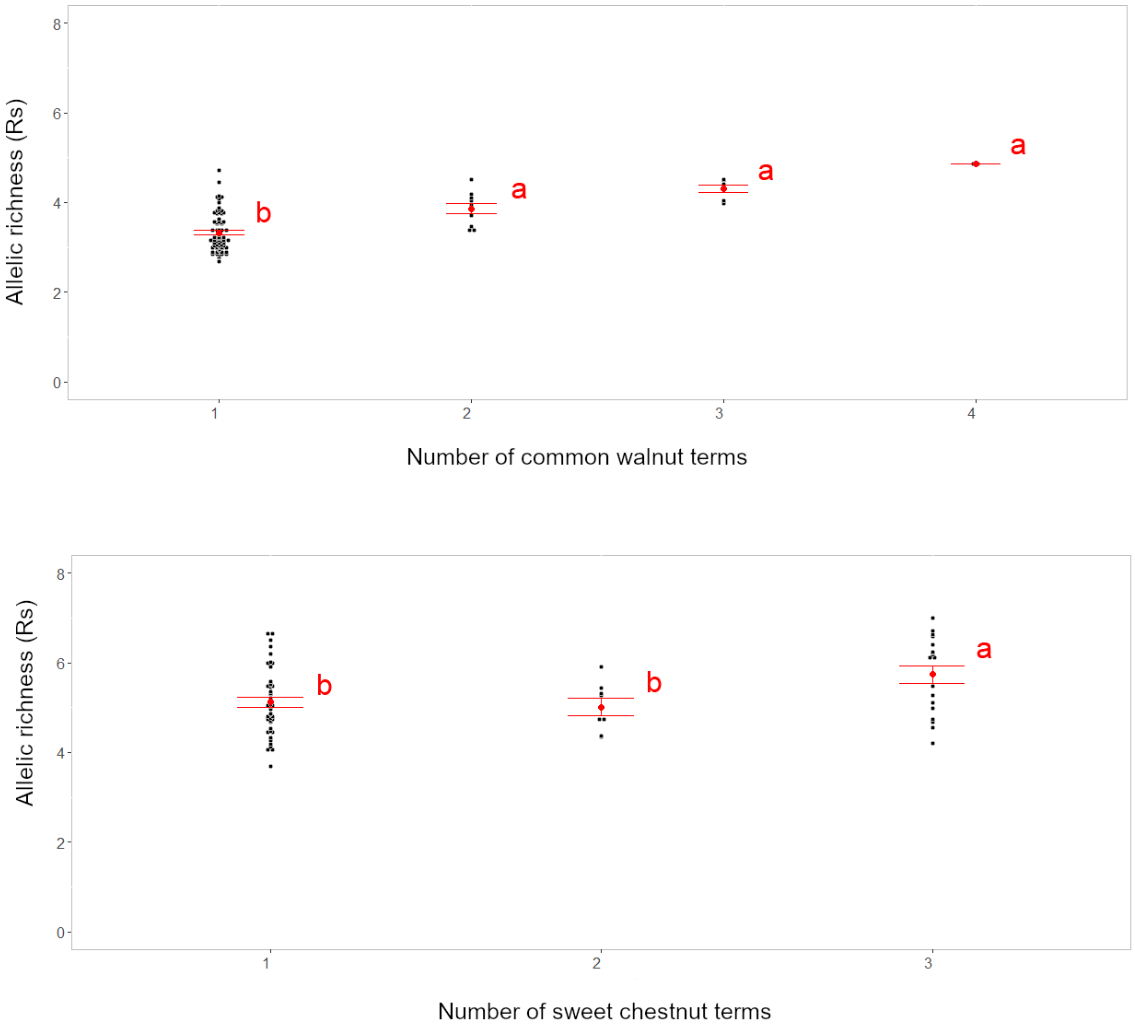

Figure S3. Dot plots comparing allelic richness (*Rs*) of common walnut and sweet chestnut populations computed using SSR markers and number of linguistic terms commonly used to design *J. regia* and *C. sativa* in the sampling sites. Mean (red dot) and standard error (red bar) are reported. Mean values showing the same letter were not significantly different at $P \geq 0.05$ according to the non-parametric post hoc Dunn's test.

References Figures

Gordon, R.G. (2005). Ethnologue: Languages of the World, Fifteenth Edition. Dallas, TX: SIL International. Retrieved from http://www.Ethnologue.com/.

Jost, L. (2008). G(ST) and its relatives do not measure differentiation. *Molecular Ecology, 17*(18), 4015–4026. doi: 10.1111/j.1365-294x.2008.03887.x.

Krebs, P., Pezzatti, G.B., Beffa, G., Tinner, W., Conedera, M. (2019). Revising the sweet chestnut (*Castanea sativa* Mill.) refugia history of the last glacial period with extended pollen and macrofossil evidence. *Quaternary Science Reviews, 206*, 111-128. doi.org/10.1016/j.quascirev.2019.01.002.

Mattioni, C., Martin, M.A., Chiocchini, F., Cherubini, M., Gaudet, M., Pollegioni,…Villani F. (2017). Landscape genetics structure of European sweet chestnut (*Castanea sativa* Mill): indications for conservation priorities. *Tree Genetics and Genomes, 13*, 39. doi:10.1007/s11295-017-1123-2.

Pollegioni, P., Woeste, K., Chiocchini F., Del Lungo S, Ciolfi M., Olimpieri I.,…Malvolti M.E. (2017). Rethinking the history of common walnut (*Juglans regia* L.) in Europe: Its origins and human interactions. *PLoS ONE, 12*(3):e0172541. doi.org/10.1371/journal.pone.0172541.

Table S1. Description of 91 common walnut populations (Pollegioni et al., 2017) and 73 sweet chestnut populations (Mattioni et. al., 2017) sampled in Eurasia. Number of samples (N), and geographic description for 91 common walnut and 73 sweet chestnut populations collected across the species' Eurasian range. Language name, subgroup, family and phylum spoken by human communities for each geographic sampling site were also reported according to The Ethnologue website (Gordon, 2005).

Country	Province	Population	Abbreviation	N	Language	Subgroup	Family	Phylum
Common walnut populations								
Kyrgyzstan	Jalal-Abad	Ak-Terek	TEREK	45	Kyrgyz	Kipchak	Turkic	Altaic
		Sharap	SHARAP	18	Kyrgyz	Kipchak	Turkic	Altaic
		Yaradar	YARADAR	16	Kyrgyz	Kipchak	Turkic	Altaic
		Shaidan	SHAIDAN	24	Kyrgyz	Kipchak	Turkic	Altaic
		Kyzyl-Ungur	KYZYL	45	Kyrgyz	Kipchak	Turkic	Altaic
		Katar-Yangak	KATAR	19	Kyrgyz	Kipchak	Turkic	Altaic
		Kyok-Sarau	KYOK	25	Kyrgyz	Kipchak	Turkic	Altaic
		Kyr-sai	KYR	27	Kyrgyz	Kipchak	Turkic	Altaic
		Ters-Kolt	TERS	24	Kyrgyz	Kipchak	Turkic	Altaic
Uzbekistan	Namangan	Kamchik	KAMCHIK	18	Uzbek	Karluk	Turkic	Altaic
	Fergana	Yakkatut	YAKKATUT	18	Uzbek	Karluk	Turkic	Altaic
	Tashkent	Sidjak	SIDJAK	10	Uzbek	Karluk	Turkic	Altaic
		Charvak	CHARVAK	18	Uzbek	Karluk	Turkic	Altaic
		Nanai	NANAI	18	Uzbek	Karluk	Turkic	Altaic
		Bogustan	BOGUSTAN	20	Uzbek	Karluk	Turkic	Altaic
		Bostanlyk	BOSTANLIK	18	Uzbek	Karluk	Turkic	Altaic
		Karankul	KARANKUL	36	Uzbek	Karluk	Turkic	Altaic
	Surkhandarya	Djarkurgan	DJARKU	16	Uzbek	Karluk	Turkic	Altaic
	Jizakh	Bakhmal	BAKHMAL	15	Tajiki	Western	Iranian	Indo-European i
					Uzbek	Karluk	Turkic	Altaic
		Farish	FARISH	19	Uzbek	Karluk	Turkic	Altaic
		Andigen	ANDIGEN	12	Uzbek	Karluk	Turkic	Altaic
		Katta-Bogdan	KATTA	38	Uzbek	Karluk	Turkic	Altaic
		Khayat	KHAYAT	16	Uzbek	Karluk	Turkic	Altaic
		Yamchi	YAMCHI	10	Uzbek	Karluk	Turkic	Altaic
		Karri	KARRI	20	Uzbek	Karluk	Turkic	Altaic
		Madjerum	MADJERUM	28	Uzbek	Karluk	Turkic	Altaic

China	Xinjiang	Gongliu-1	GUILI-1	34	Uyghur	Karluk	Turkic	Altaic
					Chinese, Mandarin	-	Sinitic	Sino-Tibetan
		Gongliu-2	GUILI-2	67	Uyghur	Karluk	Turkic	Altaic
					Chinese, Mandarin	-	Sinitic	Sino-Tibetan
		Gongliu-3	GUILI-3	27	Uyghur	Karluk	Turkic	Altaic
					Chinese, Mandarin	-	Sinitic	Sino-Tibetan
		Urumqi	URUMQI	29	Uyghur	Karluk	Turkic	Altaic
					Chinese, Mandarin	-	Sinitic	Sino-Tibetan
	Shandong	Sunbè	SUNBE	19	Chinese, Mandarin	-	Sinitic	Sino-Tibetan
	Tibet	Dashuicun	DASH	48	Tibetan	Western	Tibeto-Burman	Sino-Tibetan
					Chinese, Mandarin	-	Sinitic	Sino-Tibetan
Pakistan	Gilgit-Baltistan	Gilgit Valley	GILGIT	21	Urdu	Central	Indo-Aryan	Indo-European
		Hunza Valley	HUNZA	25	Urdu	Central	Indo-Aryan	Indo-European
Tajikistan	Karotegin	Shouli	SHOULI	16	Tajiki	Western	Iranian	Indo-European
Iran	Alborz	Karaj	KARAJ	12	Persian, Iranian	Western	Iranian	Indo-European
Georgia	Kakheti	Lagodekh	LAGO	15	Georgian	-	Karto-Zan	Kartvelian
	Shida Kartli	Skra	SKRA	21	Georgian	-	Karto-Zan	Kartvelian
Turkey		Anatolia	ANATOLIA	19	Turkish	Southern	Turkic	Altaic
					Luvial a	-	Anatolian	Indo-European
		Trabzon	TRABZON	22	Turkish	Southern	Turkic	Altaic
					Luvial a	-	Anatolian	Indo-European
Greece	Macedonia	Paiko_A	PAIKO-A	24	Greek	Attic	Hellenic	Indo-European
	Macedonia	Paiko_B	PAIKO-B	27	Greek	Attic	Hellenic	Indo-European
	Peloponnese	Arcadia	ARCADIA	20	Greek	Attic	Hellenic	Indo-European
	West Crete	Chania	CHANIA	33	Greek	Attic	Hellenic	Indo-European
Romania	Transylvania	Brasov	BRASOV	26	Romanian	Eastern-Romance	Italic	Indo-European
Moldova	Chișinău	Chișinău	CHISINAU	34	Romanian	Eastern-Romance	Italic	Indo-European
Hungary	Bács-Kiskun	Ctsatalja	CTSATALJA	30	Hungarian	-	Finno-Ugric	Uralic
		Melykut	MELYKUT	32	Hungarian	-	Finno-Ugric	Uralic
	Baranya	Pecs	PECS	31	Hungarian	-	Finno-Ugric	Uralic
	Fejér	Dunava	DUNAVA	20	Hungarian	-	Finno-Ugric	Uralic
	Szabolcs-Szatmár-Bereg	Milota	MILOTA	20	Hungarian	-	Finno-Ugric	Uralic
		Nagyar	NAGYAR	30	Hungarian	-	Finno-Ugric	Uralic
		Tiszakorod	TISZAKOROD	29	Hungarian	-	Finno-Ugric	Uralic
		Vasarosnameny	VASARO	20	Hungarian	-	Finno-Ugric	Uralic

	Györ-Moson-Sopron	Bony	BONY	38	Hungarian	-	Finno-Ugric	Uralic
		Mosonmagyar	MOSONM	20	Hungarian	-	Finno-Ugric	Uralic
Slovakia	Nitra	Nová Dedina	DEDINA	34	Slovak	Western Slavic	Balto-Slavic	Indo-European
France	Centre	Orleans	ORLEAN	16	French	Western Romance	Italic	Indo-European
	Poitou-Charentes	Poitiers	POITIERS	31	French	Western Romance	Italic	Indo-European
	Auvergne	Puy-de-Dome	PUYDOME	45	French	Western Romance	Italic	Indo-European
	Rhone-Alpes	Chambéry	CHAMBERY	44	French	Western Romance	Italic	Indo-European
Spain	Catalogna	Girona	GIRONA	20	Spanish	Western Romance	Italic	Indo-European
Italy	Veneto	Osigo	OSIGO	13	Italian	Western Romance	Italic	Indo-European
	Friuli	Pordenone	PORD	10	Italian	Western Romance	Italic	Indo-European
		Preone	PREONE	12	Italian	Western Romance	Italic	Indo-European
		Gabria	GABRIA	10	Italian	Western Romance	Italic	Indo-European
	Umbria	Castel Giorgio	GIORGIO	15	Italian	Western Romance	Italic	Indo-European
	Lazio	Palombara Sabina	SABINA	8	Italian	Western Romance	Italic	Indo-European
	Abruzzo	Pescasserolig	PESC	20	Italian	Western Romance	Italic	Indo-European
		Civitella Alfedenag	ALF	18	Italian	Western Romance	Italic	Indo-European
		Villetta Barreag	BARREA	21	Italian	Western Romance	Italic	Indo-European
	Molise	Montenero Val Cocchiaro	VALCO	10	Italian	Western Romance	Italic	Indo-European
		Rionero Sannitico	RIONERO	8	Italian	Western Romance	Italic	Indo-European
		Forlì del Sannio	SANNIO	20	Italian	Western Romance	Italic	Indo-European
		Miranda	MIRA	14	Italian	Western Romance	Italic	Indo-European
		S. Maria del Molise	FONT	20	Italian	Western Romance	Italic	Indo-European
		San Massimo	MAS	20	Italian	Western Romance	Italic	Indo-European
		Sepinoh	ALTILIA	20	Italian	Western Romance	Italic	Indo-European
	Campania	Santa Croce del Sannio	CROCE	11	Italian	Western Romance	Italic	Indo-European
		Circello	CIRCE	20	Italian	Western Romance	Italic	Indo-European
		San Marco dei Cavoti	CAVOTI	29	Italian	Western Romance	Italic	Indo-European
		San Giorgio la Molara	MOLARA	8	Italian	Western Romance	Italic	Indo-European
		Montecalvo Irpino	MONTEC	26	Italian	Western Romance	Italic	Indo-European
		Ariano Irpino	ARIANO	23	Italian	Western Romance	Italic	Indo-European
		Casolla	CASOLLA	10	Italian	Western Romance	Italic	Indo-European
		Tufino	TUFINO	10	Italian	Western Romance	Italic	Indo-European
		San Michele Serino	SERINO	10	Italian	Western Romance	Italic	Indo-European
		Montella	MONT	20	Italian	Western Romance	Italic	Indo-European
	Sicily	Ragusa	RAGUSA	10	Italian	Western Romance	Italic	Indo-European

		Anapo Valley	ANAPO	10	Italian	Western Romance	Italic	Indo-European
		Bivona	BIVONA	10	Italian	Western Romance	Italic	Indo-European
Total		-		2008				
Sweet chestnut populations								
Portugal	Bragança District	Bragança	PT01	17	Portuguese	Western Romance	Italic	Indo-European
	Vila Real	Vila Pouca	PT02	20	Portuguese	Western Romance	Italic	Indo-European
	Guarda District	Guarda	PT03	20	Portuguese	Western Romance	Italic	Indo-European
Spain	Andalucia	Bubión	SP01	14	Spanish	Western Romance	Italic	Indo-European
		Gaucín	SP04	26	Spanish	Western Romance	Italic	Indo-European
		Güejar Sierra	SP05	14	Spanish	Western Romance	Italic	Indo-European
		Paterna	SP09	14	Spanish	Western Romance	Italic	Indo-European
		Pujerra	SP11	12	Spanish	Western Romance	Italic	Indo-European
		Santa Elena	SP12	11	Spanish	Western Romance	Italic	Indo-European
		Sierra Norte	SP14	15	Spanish	Western Romance	Italic	Indo-European
		Trasierra	SP17	35	Spanish	Western Romance	Italic	Indo-European
	Cataluña	Castanyet	SP02	15	Spanish	Western Romance	Italic	Indo-European
		Prades	SP10	15	Spanish	Western Romance	Italic	Indo-European
		Viladrau	SP16	15	Spanish	Western Romance	Italic	Indo-European
	Extremadura	Hervas	SP06	14	Spanish	Western Romance	Italic	Indo-European
		Valverde	SP15	15	Spanish	Western Romance	Italic	Indo-European
	Castilla-León	Médulas	SP08	29	Spanish	Western Romance	Italic	Indo-European
	Asturias	Mieres	SP07	15	Spanish	Western Romance	Italic	Indo-European
	Galicia	Costa Atlántica	SP03	21	Spanish	Western Romance	Italic	Indo-European
		Sierra Faro	SP13	23	Spanish	Western Romance	Italic	Indo-European
France	Aquitania	Dordogne	FR01	15	French	Western Romance	Italic	Indo-European
		Pyrénées Atlantiques	FR03	9	French	Western Romance	Italic	Indo-European
	Midi-Pyrénées	Aveyron	FR02	16	French	Western Romance	Italic	Indo-European
England	Gloucestershire	Speech House	UK01	20	English	Anglo-Frisian	Germanic	Indo-European
Italy	Sicilia	Madonie	IT01	26	Italian	Western Romance	Italic	Indo-European
	Calabria	Sila Piccola	IT02	26	Italian	Western Romance	Italic	Indo-European
	Basilicata	Mt. Vulture	IT03	25	Italian	Western Romance	Italic	Indo-European
	Marche	Mt. Laga	IT04	26	Italian	Western Romance	Italic	Indo-European
	Lazio	Mt. Cimini-	IT05	23	Italian	Western Romance	Italic	Indo-European
	Toscana	Mugello	IT06	19	Italian	Western Romance	Italic	Indo-European
	Piemonte	Trontano	IT07	26	Italian	Western Romance	Italic	Indo-European

	Piemonte	V. Pellice	IT08	26	Italian	Western Romance	Italic	Indo-European
	Friuli	V. del Natisone	IT09	26	Italian	Western Romance	Italic	Indo-European
Slovakia	Banska Bystrica	Modrý Kameň	SK01	27	Slovak	Western Slavic	Balto-Slavic	Indo-European
	Nitra	Jelenec	SK02	22	Slovak	Western Slavic	Balto-Slavic	Indo-European
	Bratislava	Častá	SK03	20	Slovak	Western Slavic	Balto-Slavic	Indo-European
	Bratislava	Bratislava	SK04	13	Slovak	Western Slavic	Balto-Slavic	Indo-European
	Bratislava	Modra	SK05	10	Slovak	Western Slavic	Balto-Slavic	Indo-European
Hungary	Pecs	Nagymasros	HU01	30	Hungarian	-	Finno-Ugric	Uralic
Bulgaria	Blagoevgrad	Belasitsa	BU01	50	Bulgarian	South Slavic	Balto-Slavic	Indo-European
	Blagoevgrad	Slavyanka	BU02	21	Bulgarian	South Slavic	Balto-Slavic	Indo-European
	Blagoevgrad	North west Pirin	BU03	20	Bulgarian	South Slavic	Balto-Slavic	Indo-European
	Blagoevgrad	South west Pirin	BU04	21	Bulgarian	South Slavic	Balto-Slavic	Indo-European
	Blagoevgrad	Ograjden	BU05	42	Bulgarian	South Slavic	Balto-Slavic	Indo-European
	Montana	Western Stara	BU06	21	Bulgarian	South Slavic	Balto-Slavic	Indo-European
Romania	Dobresti	Bihor	RO01	11	Romanian	Eastern-Romance	Italic	Indo-European
	Baia Sprie	Maramures	RO02	11	Romanian	Eastern-Romance	Italic	Indo-European
	Tarnita	Mehedinti	RO03	12	Romanian	Eastern-Romance	Italic	Indo-European
Greece	S-E-Macedonia	Holomontas	GR01	26	Greek	Attic	Hellenic	Indo-European
	C-Macedonia	Hortiatis	GR02	26	Greek	Attic	Hellenic	Indo-European
	W-Macedonia	Dafni	GR03	26	Greek	Attic	Hellenic	Indo-European
	N-Macedonia	Paiko	GR04	26	Greek	Attic	Hellenic	Indo-European
Turkey	Duzce	Akcakoca	TR01	24	Turkish	Southern	Turkic	Altaic
					Luvial a	-	Anatolian	Indo-European
	Sinop	Sinop	TR02	31	Turkish	Southern	Turkic	Altaic
					Luvial a	-	Anatolian	Indo-European
	Artvin	Hopa	TR03	22	Turkish	Southern	Turkic	Altaic
					Luvial a	-	Anatolian	Indo-European
	Trabzon	Meryem Ana	TR04	30	Turkish	Southern	Turkic	Altaic
					Luvial a	-	Anatolian	Indo-European
	Giresun	Giresun	TR05	26	Turkish	Southern	Turkic	Altaic
					Luvial a	-	Anatolian	Indo-European
	Sakarya	Karadere	TR06	21	Turkish	Southern	Turkic	Altaic
					Luvial a	-	Anatolian	Indo-European
	Kocaeli	Sardala	TR07	23	Turkish	Southern	Turkic	Altaic
					Luvial a	-	Anatolian	Indo-European

	Yalova	Cinarcik	TR08	26	Turkish	Southern	Turkic	Altaic
					Luvial [a]	-	Anatolian	Indo-European
	Kocaeli	Golcuk	TR09	25	Turkish	Southern	Turkic	Altaic
					Luvial [a]	-	Anatolian	Indo-European
	Sakarya	Sapanca	TR10	24	Turkish	Southern	Turkic	Altaic
					Luvial [a]	-	Anatolian	Indo-European
	Bursa	Bursa	TR11	25	Turkish	Southern	Turkic	Altaic
					Luvial [a]	-	Anatolian	Indo-European
	Canakkale	Bayramic	TR12	31	Turkish	Southern	Turkic	Altaic
					Luvial [a]	-	Anatolian	Indo-European
	Izmir	Kemalpasa	TR13	23	Turkish	Southern	Turkic	Altaic
					Luvial [a]	-	Anatolian	Indo-European
	Manisa	Demirci	TR14	22	Turkish	Southern	Turkic	Altaic
					Luvial [a]	-	Anatolian	Indo-European
Georgia	Shida Kartli	Rikoti Pass	GE01	31	Georgian	-	Karto-Zan	Kartvelian
	Adjara	Uchkhiti	GE02	30	Georgian	-	Karto-Zan	Kartvelian
	Adjara	Mtirala National Park	GE03	26	Georgian	-	Karto-Zan	Kartvelian
	Abkhasia	Ochamchira	GE04	14	Georgian	-	Karto-Zan	Kartvelian
	Abkhasia	Tkwarchelsk	GE05	15	Georgian	-	Karto-Zan	Kartvelian
Azerbaijan	Oguz	Oguz	AZ01	32	Azeri Turkish	Oghuz	Turkic	Altaic
Russia	Krasnodarskiy Kray	Chvigepse	RU01	28	Russian	East Slavic	Balto-Slavic	Indo-European
					Georgian	-	Karto-Zan	Kartvelian
Total		-		1608				

[a] Luvial is an ancient language spoken in a vast area stretching from the Sakarya river basin in northwestern Anatolia to the Euphrates valley in present-day Syria during second and first millennia BC.

Table S2. Linguistic terms denoting common walnut in the Sino-Dené-Caucasian (Basque, Proto_Burushaski, Proto-North Caucasian and Sino-Tibetan) Afro-Asiatic and Eurasiatic (Dravidian, Kartvelian, Altaic, Indo-European and Uralic) language Super_Philum of Eurasia.

Super_Phylum	Phylum	Family	Language	Walnut	Reference [a]
Sino-Dene-Caucasian<br>**HwV́rλ̣̄V* (nut, seed)<br>**ḳV̆rḳV̆̄* (nut)<br>**ṭVłV* (egg)	Basque<br>**hur̄* (walnut, hazelnut)<br>**kankano* (large kernel)		Basque	*intxaur*<br>*kankano* (nut)	LWED
	Proto-Burushaski<br>**khakhā́jo* (walnut)<br>**tile* (walnut)		Burushaski	*khakhā́jo*<br>*tili*	LWED
	Sino-Tibetan<br>**lĭ* (fruit, seed)<br>**t[i]l* (egg, testicle)				LWED
		Sinitic	Mandarin-Chinese	**lit* (fruit)<br>*d(h)rəj* (ant's egg)<br>*hú táo*	LWED<br>Laufer, (1919);<br>Thompson, (1961)
		Tibetic	Tibetan	*li* (apple)<br>*thul* (egg, testicle)<br>*star-ka*<br>*kara*	LWED<br>Laufer (1919)<br>Weckerle, Huber, Yongping, & Weibang (2005).
	North Caucasian<br>*ʔwā̄rλ̣̄_V (~ -ō-,-Ł-) (nut)<br>**ḳV̆rḳV̆(-nV)* (small stone, grain, egg)				LWED
		West-Caucasian<br>**ĺa* (walnut)<br>**ḳanəḳa / *ḳaḳanə* (nut, egg)	Abaza	*ra.sa*<br>*ḳaḳan* (nut, egg)	LWED
Afro-Asiatic	Afro-Asiatic				
		Semitic	Akkadian<br>Ugaritic<br>Hebrew<br>Aramaic	*gūzu*<br>*'rgz*<br>*‘egoz*<br>*‘egoza*	Tóth, (2007)<br>Pardee, (2001)<br>Borbor (2010); Laufer (1919)<br>Borbor (2010); Laufer (1919)

			Arabic	*jauz*	Borbor (2010); Laufer (1919)
			Syriac	*gauza*	Sokoloff (2009)
Eurasiatic<br>**pVč`V* (seed)<br>**KVrV, *ŋuńV* (nut)	Dravidian<br>**kur*- (nut)<br>**nuŋ*- (nut)				LWED
			Tamil	*Akhrot*<br>*naṭṭu* (nut)<br>*kuru* (nut)	LWED<br>Borbor (2010); Laufer (1919)
	Kartvelian<br>**ḳaḳ-al*- (nut)	Karto-Zan	Svan	*gak'*	LWED<br>Borbor (2010); Laufer (1919)
			Georgian	*ḳaḳali*<br>*ni-gosi/goz-*	LWED<br>Borbor (2010); Laufer (1919)
	Altaic<br>**pisV (~ p`-, -i̯a-)* (seed)<br>**kȍru (~ -ŕ-)* (nut)<br>**ńaŋo* (nut)				LWED
		Turkic<br>**jAŋgak* (walnut)			Dwyer (2007)
			Kyrgyz	*ǯaŋaq or ǯaŋɣaq*	Dwyer (2007)
			Northern Uzbek	*jɔŋɣɔq*	Dwyer (2007)
			Uyghur	*jaŋɣaq*	Dwyer (2007)
			Turkish	*koz*<br>*ceviz ağacı*	Borbor (2010); Laufer (1919)<br>Vahdati (2014)
			Azeri Turkish	*qoz*<br>*əkin noxudu* (pea)	Borbor (2010); Laufer (1919)<br>Mikić (2018)
	Indo-European<br>**kar*- (nut, hazelnut)<br>**khneu*- (nut)				LWED
		Anatolian	Hittite	*arra* (walnut)<br>*harau*- (poplar)	Peza and Peza (2014)
		Hellenic<br>*(k)árüo-n* (walnut, hazelnut)			LWED<br>Gamkrelidze and Ivanov (1995)
			Greek	*karydiá*<br>*karia basilica* (royal nut)	Gamkrelidze and Ivanov (1995)
		Proto-Armenian<br>*k:ak:al* (walnut)			

	Armenian	*k’ak’al*<br>*en-goyz*	Borbor (2010); Laufer (1919)
Proto-Albanian<br>*arrë* (walnut)			Peza and Peza (2014)<br>Gamkrelidze and Ivanov (1995)
	Albanian	*arrë*<br>*nyç* (gnarl)	www.wordhippo.com
Baltic<br>**reîš-a-* (walnut)			Gamkrelidze and Ivanov (1995)
	Latvian	*riekstkoks*	www.wordhippo.com
	Lithuanian	*graikinis riešutas*	www.wordhippo.com
Slavic<br>**orēxъ* (walnut)			LWED<br>Gamkrelidze and Ivanov (1995)
	Slovak	*orech*	www.wordhippo.com
	Belarusian	*грэцкі арэх*	www.wordhippo.com
	Bulgarian	*орех*	www.wordhippo.com
	Croatian	*orah*	www.wordhippo.com
	Czech	*ořech*	www.wordhippo.com
	Macedonian	*орев*	www.wordhippo.com
	Polish	*orzech włoski*	www.wordhippo.com
	Russian	*орех*	www.wordhippo.com
	Serbian	*орах*	www.wordhippo.com
	Slovenian	*oreh*	www.wordhippo.com
	Ukrainian	*volos'kyy horikh*	www.wordhippo.com
Germanic<br>**xnut-* (nut)			LWED<br>Gamkrelidze and Ivanov (1995)
	Danish	*valnød*	www.wordhippo.com
	Dutch	*walnoot*	www.wordhippo.com
	Old English	*walhnutu*	Gamkrelidze and Ivanov (1995)
	German	*walnuss*	www.wordhippo.com
	Norwegian	*valnøtt*	www.wordhippo.co+m
	Swedish	*valnöt*	www.wordhippo.com
Celtic			LWED
	Welsh	*cneuen*	Gamkrelidze and Ivanov (1995)
	Old Irish	*knot*<br>*curar* (pignut)	Gamkrelidze and Ivanov (1995)<br>LWED
	Breton	*keler* (earth chestnut)	LWED
Italic			LWED
	Latin	*nux* (walnut)<br>*carina* (boat, half	LWED<br>Gamkrelidze and Ivanov (1995)

		walnut shell)<br>*Juglans* (Jupiter's acorn)	
	Italian	*noce*<br>*carena* (boat)	LWED
	French	*noyer*<br>*coque* (boat)	LWED
	Spanish	*nuez*<br>*cáscara* (boat)	LWED
	Romanian	*nuc*<br>*carenă* (boat)<br>*orech*	LWED
	Portuguese	*noz*<br>*casco* (boat)	LWED
	Sardinian	*nughe*<br>*carena* (boat)	LWED
	Catalan	*nou*<br>*casc* (boat)	LWED
	Galician	*nogueira*<br>*casco* (boat)	LWED
Indo-Iranian<br>**a-/an-gōza*<br>(something hidden inside a shell)<br>*hud* (nut)			Borbor (2010); Laufer (1919)
	Old Persian	*gawz*<br>*nohud* (pea)	Borbor (2010); Hasandust (2000)<br>Mikić (2018)
	Parthian	*ngwz-*	Borbor (2010); Hasandust (2000)
	Sogdian	*γwš*	Borbor (2010); Hasandust (2000)
	Modern Persian	*gôz*<br>*gerdoo*<br>*nxud frngi* (pea)	Borbor (2010); Hasandust (2000)<br>Mikić, (2018)
	Tajik	*gôz*<br>*cormacz*<br>*naxūddona* (pea)	Borbor (2010); Hasandust (2000)<br>Baizoyev and Hayward (2003)<br>Mikić, (2018)
	Ossetian	*än-gũz*	Borbon (2010)
Indo-Aryan<br>*ak-ṣōṭa-*	Sanskrit	*AkhoTa*<br>*karaka* (cocoa nut	Borbor (2010); Hasandust (2000)<br>LWED

				shell)	
			Urdu	*akhrot*	Borbor (2010); Hasandust (2000)
			Hindi	*akhrot*	Borbor (2010); Hasandust (2000)
			Kasmiri	*doon*	Vahdati (2014)
	Uralic **päškV*				LWED
		Finno-Ugric			
			Finnish	*saksan pänkinä* (German nut)	www.wordhippo.com
			Hungarian	*dió*	Tóth (2007)

[a] The acronyms of The Languages of the World Etymological Database, part of the Tower of Babel project is LWED

Table S3. Linguistic terms denoting sweet chestnut in the Sino-Dené-Caucasian (Basque) Afro-Asiatic and Eurasiatic (Dravidian, Kartvelian, Altaic, Indo-European and Uralic) language Super_Philum of Eurasia.

**Super_Phylum**	**Phylum**	**Family**	**Language**	**Chestnut**	**Reference** [a]
Sino-Dene-Caucasian<br>**q̇wăłV́* (bark, skin)	Proto-Basque<br>**kVl* (chestnut shell)		Basque	*koskol* (chestnut shell, husk)<br>*gaztaina*	LWED
	Proto-North Caucasian<br>**q̇wăłV* (bark, crust)		Beztha<br>Abkhazian	*q̇eq̇el-ba* (bark)<br>*á-xja*	LWED<br>Spruit (1985)
Afro-Asiatic	Afro-Asiatic				
		Semitic<br>**blwt*	Akkadian	*ballutu* (chestnut/oak)	De Lafayette (2013)
			Aramaic	*balut* (chestnut/oak)	De Lafayette (2013)
			Arabic	*ballūṭ* (oak, acorn)<br>*kastanāʾ* (chestnut)	De Lafayette (2013)
			Hebrew	*ʿarmon* (chestnut/plane tree)	Gill (2010)
Eurasiatic<br>**bVrq̇wV* (edible fuit)<br>**dʷirV* (tree, wood)	Proto-Kartvelian<br>**berq̇en-* (wild pear)<br>**dwire* (log, beam)<br>**ć̣ab-*				LWED
		Karto-Zan	Georgian	*berq̇ena* (wild plum)<br>*dire* (log, beam)<br>*c̣abli*	LWED
	Proto-Altaic<br>**t`i̯ór(g)e* (support, beam)				
		Turkic<br>**terki* (table)	Turkish	*kestane*<br>*bayat fıkra*	Cooper (2010)
			Azeri Turkish	*şabalıd*	Wall and Aghayeva (2014)
	Proto-Indo-European<br>**kastAno-*<br>**derw-* (tree, oak, wood)<br>**bhrūg-* (fruit)				Huld (2012)<br>Cooper (2010)<br>LWED
		Proto-Anatolian<br>**tő́ru-* (wood, tree)			Cooper (2010)
			Hittite†	*taru* (tree)	LWED

	Anatolian	*dōru* (oak, chestnut)	LWED
Hellenic<br>*kástanon*<br>*drũs* (oak)			Huld (2012)<br>Cooper (2010)
	Greek	*kástano*<br>*dios balanos* (divine acorn)<br>*karua Euboikè* (Euboeian nut)<br>*drys* (oak)<br>*froúto* (fruit)	Huld (2012)<br>LWED
Proto-Armenian<br>**kask*			Huld (2012)
	Armenian	*šaganak*	Huld (2012)
Proto-Albanian<br>*dru* (wood, log)			Cooper (2010)<br>LWED
	Albanian	*gështenjë*<br>*drusk* (oak)<br>*pāfrujt* (fruitless)	https://en.wiktionary.org/wiki/chestnut<br>LWED
Baltic<br>**késten*<br>**der̃w-â* (resinous wood)			LWED
	Latvian	*kastanis*<br>*darva* (tar)	Cooper (2010)<br>LWED
	Lithuanian	*kaštonas*<br>*derva* (tar)	Cooper (2010)<br>LWED
Slavic<br>**késten*<br>**dervo* (tree)			LWED
	Slovak	*gaštan*<br>*drevo* (tree)	Cooper (2010)<br>LWED
	Belarusian	*kaštán*<br>*dzjérava* (tree)	Cooper (2010)<br>LWED
	Bulgarian	*késten*<br>*dărvó* (tree)	Cooper (2010)<br>LWED
	Croatian	*kesten*<br>*dȑvo* (tree)	Cooper (2010)<br>LWED
	Czech	*kaštan*<br>*dřevo* (wood)	Cooper (2010)<br>LWED

	Macedonian	*kósten*<br>*drvo* (tree)	Cooper (2010)<br>LWED
	Polish	*kasztan*<br>*drzewo* (tree)	Cooper (2010)<br>LWED
	Russian	*kaštán*<br>*dérevo* (tree)	Cooper (2010)<br>LWED
	Serbian	*kesten*<br>*dȑvo* (tree)	Cooper (2010)<br>LWED
	Slovenian	*kostanj*<br>*drevọ̑* (tree)	Cooper (2010)<br>LWED
	Ukrainian	*kaštán*<br>*dérevo* (tree)	Cooper (2010)<br>LWED
Germanic<br>*kastan(i)e*<br>**tri(w)u* (tree)<br>*fruht* (fruit)			Cooper (2010)<br>LWED
	Danish	*kastanje*<br>*trä* (tree)<br>*frugt* (fruit)	Cooper (2010)<br>LWED
	Dutch	*kastanje*<br>*teer* (tar)<br>*vrucht (fruit)*	Cooper (2010)<br>LWED
	Old English	*chesten*<br>*trēow* (tree)<br>*frute* (fruit)	Cooper (2010)<br>LWED
	German	*kastanie*<br>*teer* (tar)<br>*frucht* (fruit)	Cooper (2010)<br>LWED
	Norwegian	*kastanje*<br>*tre* (tree)<br>*frukt* (fruit)	Cooper (2010)<br>LWED
	Swedish	*kastanj*<br>*träd* (tree)<br>*frukt* (fruit)	Cooper (2010)<br>LWED
Celtic<br>*castan*<br>**daru, *derwā* (oak)<br>*diffrwyth* (fruitless)			Cooper (2010)<br>LWED
	Welsh	*castan*	Cooper (2010)

		derw-en (oak)	LWED
	Old Irish	*castán*<br>*daur* (oak)	Cooper (2010)<br>LWED
	Breton	*kistin*<br>*dervenn* (oak)<br>*frouezh* (fruit)	Cooper (2010)<br>LWED
Italic/Latin<br>*castanea*<br>*dūrus* (hard)<br>*fructus* (fruit)			Cooper (2010)
	Italian	*castagna*<br>*duro* (hard)<br>*frutto* (fruit)	https://en.wiktionary.org/wiki/chestnut<br>LWED
	French	*châtaigne*<br>*dur* (hard)<br>*fruit* (fruit)	https://en.wiktionary.org/wiki/chestnut<br>LWED
	Spanish	*castaña*<br>*duro* (hard)<br>*fruto* (fruit)	https://en.wiktionary.org/wiki/chestnut<br>LWED
	Romanian	*castană*<br>*dur* (hard)<br>*fruct* (fruit)	https://en.wiktionary.org/wiki/chestnut<br>LWED
	Portuguese	*castanha*<br>*duro* (hard)<br>*fruta* (fruit)	https://en.wiktionary.org/wiki/chestnut<br>LWED
	Sardinian	*castànza*<br>*duru* (hard)<br>*frutu* (fruit)	https://en.wiktionary.org/wiki/chestnut<br>LWED
	Catalan	*castanya*<br>*duro* (hard)<br>*frutto* (fruit)	https://en.wiktionary.org/wiki/chestnut<br>LWED
	Galician	*castaña*<br>*duro* (hard)<br>*froita* (fruit)	https://en.wiktionary.org/wiki/chestnut<br>LWED
Indo-Iranian<br>**blwt'*			
	Old Persian	*shâh-balūṭ*<br>*kastana* (tree)	Laufer (1919)
	Sanskrit	*kashta* (tree)	Cooper (2010)

			drus (tree)	LWED
	Finno-Ugric			
		Finnish	*kastanja*	https://en.wiktionary.org/wiki/chestnut
		Hungarian	*gesztenye*	https://en.wiktionary.org/wiki/chestnut

[a] The acronyms of The Languages of the World Etymological Database, part of the Tower of Babel project is LWED

References Tables

Baizoyev, A., & Hayward, J. (2003). A Beginners' Guide to Tajiki. London, Routledge Press.
Borbor, D. (2010). A syntacto-cognitive study of the diachrony, synchrony, etymology and gloss of the New Persian format am/an*. *Journal of Persianate Studies. 3,* 222-238.
Cooper, B., (2010). Russian words for forest trees: a lexicological and etymological study. *ASEES*, *24*(1-2): 41-71.
De Lafayette, M. (2013) *Vol.1. Ancient and modern Aramaic Assyrian Syriac-English dictionary*. New York Berlin. Time Square Press.
Dwyer, A.M. (2007). *Salar - a study in Inner Asian language contact processes. Part I: Phonology*. Turcologica 37, 1. Harrassowitz.
Gamkrelidze, T.V., & Ivanov, V.V. (1995). Indo-European and the Indo-Europeans: A Reconstruction and Historical Analysis of a Proto-Language and Proto-Culture. Part I: The Text. Trends in Linguistics. Studies and Monographs [TiLSM], De Gruyter Mouton.
Gill, M. (2010). Encyclopedia of Jewish Food. Hoboken, New Jersey. John Wiley & Sons Inc.
Hasandust, M. (2000). On the etymology of two Persian words: ruze and gows. *Nameye Farhangestan*, *4,* 135–139.
Hurd, M.E. (2012). An Old (but reconstructible) chestnut. *The Journal of Indo-European Studies*, *40*(3&4), 344-364.
Laufer, B. (1919). Sino-Iranica. Chinese contributions to the history of civilization in ancient Iran. *Field Museum of Natural History: 1909). Anthropological series, 15*(3), 185–530.
Mattioni, C., Martin, M.A., Chiocchini, F., Cherubini, M., Gaudet, M., Pollegioni,…Villani F. (2017). Landscape genetics structure of European sweet chestnut (*Castanea sativa* Mill): indications for conservation priorities. *Tree Genetics and Genomes, 13,* 39. doi:10.1007/s11295-017-1123-2.
Mikić, A. (2018). *Lexicon of Pulse Crops.* New York, Taylor & Francis Group, LLC.
Pardee, D. (2001). Ugaritic Science. In Daviau, P.M.M., Weigl, M., Wevers, J.W. (Eds.) *The World of the Aramaeans: Studies in Honour of Paul-Eugène Dion, Volume 3*. (pp. 223-254). Sheffield. Sheffield Accademic Press England.
Peza, L., Peza, L. (2014). Hittite language is a dialect of Pelasgian/Albanian language. *Dodona, 1*, 40-43.
Pollegioni, P., Woeste, K., Chiocchini F., Del Lungo S, Ciolfi M., Olimpieri I.,…Malvolti M.E. (2017). Rethinking the history of common walnut (*Juglans regia* L.) in Europe: Its origins and human interactions. *PLoS ONE, 12*(3):e0172541. doi.org/10.1371/journal.pone.0172541.
Sokoloff, A. (2009). Syriac lexicon: a translation from the Latin, correction, expansion, and update of C. Brockelmann's Lexicon Syriacum.(2nd ed). Winona Lake, Indiana; Piscataway, New Jersey. Eisenbrauns, Gorgias Pr Llc.
Spruit, A. (1985). Stress in Abkhaz. *Studio Caucasica*, 6, 31–81.
Thompson, K. (1961). Location and relocation of a tree crop-English Walnuts in California. *Economic Geography, 37*(2), 133-149. doi: 10.2307/141844.

Tóth, A. (2007). *Hungarian-Mesopotamian Dictionary (HMD)*. Holland. Mikes International The Hague.
Vahdati, K. (2014). Traditions and folks for walnut growing around the Silk Road. Acta Horticulturae. 1032, 19-24. doi.org/10.17660/ActaHortic.2014.1032.1.
Weckerle, C., Huber, F.K., Yongping, Y., & Weibang S. (2005). Walnuts among the Shuhi in Shuiluo, Eastern Himalayas.
Economic Botany 59(3), 287-290. https://www.jstor.org/stable/i389522
